# Effectiveness of a Novel Tablet Application in Reducing Guideline Deviations During Pediatric Cardiac Arrest: A Randomized Clinical Trial

**DOI:** 10.1001/jamanetworkopen.2023.27272

**Published:** 2023-08-03

**Authors:** Francesco Corazza, Marta Arpone, Giacomo Tardini, Valentina Stritoni, Giulia Mormando, Alessandro Graziano, Paolo Navalesi, Elena Fiorese, Sofia Portalone, Marco De Luca, Marco Binotti, Luca Tortorolo, Serena Salvadei, Alessia Nucci, Alice Monzani, Giulia Genoni, Marco Bazo, Adam Cheng, Anna Chiara Frigo, Liviana Da Dalt, Silvia Bressan

**Affiliations:** 1 Pediatric Emergency Division, Department of Women’s and Children’s Health, University Hospital of Padova, Padova, Italy; 2 Pediatric Emergency Division, Department of Women’s and Children’s Health, University of Padova, Padova, Italy; 3 Pediatric Intensive Care Unit, Department of Women’s and Children’s Health, University Hospital of Padova, Padova, Italy; 4 Emergency Medicine, Department of Medicine, University of Padova, Padova, Italy; 5 Anesthesia and Critical Care, Department of Medicine, University of Padova, Padova, Italy; 6 Meyer Simulation Centre, Meyer Children’s Hospital IRCCS, Florence, Italy; 7 Neonatal and Pediatric Intensive Care Unit, Maggiore della Carità University Hospital, University of Piemonte Orientale, Novara, Italy; 8 Pediatric Intensive Care, Istituto di Pediatria, Università Cattolica del Sacro Cuore, Rome, Italy; 9 Pediatric Emergency Medicine, Meyer Children’s Hospital IRCCS, Florence, Italy; 10 Pediatric Unit, Maggiore della Carità University Hospital, University of Piemonte Orientale, Novara, Italy; 11 Department of Pediatrics, Alberta Children’s Hospital, University of Calgary, Calgary, Canada; 12 Department of Cardiac, Thoracic, Vascular Sciences and Public Health, University of Padova, Padova, Italy; 13 Department of Emergency Medicine, Alberta Children’s Hospital, University of Calgary, Calgary, Canada

## Abstract

**Importance:**

Deviations from international resuscitation guidelines during the management of pediatric cardiac arrest are frequent and affect clinical outcomes. An interactive tablet application (app), PediAppRREST, was developed to reduce guideline deviations during pediatric cardiac arrest.

**Objective:**

To assess the effectiveness of PediAppRREST in improving the management of simulated in-hospital pediatric cardiac arrest.

**Design, Setting, and Participants:**

This multicenter 3-group simulation-based randomized clinical trial was conducted from September 2020 to December 2021 at 4 Italian university hospitals (Padua, Florence, Rome, Novara). Participants included residents in pediatrics, emergency medicine, and anesthesiology. Analyses were conducted as intention-to-treat. Data were analyzed from January to June 2022.

**Interventions:**

Teams were randomized to 1 of 3 study groups: an intervention group that used the PediAppRREST app; a control group that used a paper-based cognitive aid, the Pediatric Advanced Life Support (PALS) pocket card; and a control group that used no cognitive aids. All the teams managed the same standardized simulated scenario of nonshockable pediatric cardiac arrest.

**Main Outcomes and Measures:**

The primary outcome was the number of deviations from guidelines, measured by a 15-item checklist based on guideline recommendations. The main secondary outcomes were quality of chest compressions, team clinical performance (measured by the Clinical Performance Tool), and perceived team leader’s workload. Study outcomes were assessed via video reviews of the scenarios.

**Results:**

Overall 100 teams of 300 participants (mean [SD] age, 29.0 [2.2] years; 195 [65%] female) were analyzed by intention-to-treat, including 32 teams randomized to the PediAppRREST group, 35 teams randomized to the PALS control group, and 33 teams randomized to the null control group. Participant characteristics (210 pediatric residents [70%]; 48 anesthesiology residents [16%]; 42 emergency medicine residents [14%]) were not statistically different among the study groups. The number of deviations from guidelines was significantly lower in the PediAppRREST group than in the control groups (mean difference vs PALS control, −3.0; 95% CI, −4.0 to −1.9; *P* < .001; mean difference vs null control, −2.6; 95% CI, −3.6 to −1.5; *P* < .001). Clinical Performance Tool scores were significantly higher in the PediAppRREST group than control groups (mean difference vs PALS control, 1.4; 95% CI, 0.4 to 2.3; *P* = .002; mean difference vs null control, 1.1; 95% CI, 0.2 to 2.1; *P* = .01). The other secondary outcomes did not significantly differ among the study groups.

**Conclusions and Relevance:**

In this randomized clinical trial, the use of the PediAppRREST app resulted in fewer deviations from guidelines and a better team clinical performance during the management of pediatric cardiac arrest.

**Trial Registration:**

ClinicalTrials.gov Identifier: NCT04619498

## Key Points

**Question:**

Does the use of a tablet application cognitive aid reduce deviations from American Heart Association (AHA) resuscitation guidelines and improve the management of pediatric cardiac arrest?

**Findings:**

In this randomized clinical trial including 300 participants in 100 teams, the use of an interactive tablet application led to fewer deviations from AHA guidelines and better team performance compared with use of the AHA pocket reference card or no cognitive aid.

**Meaning:**

This randomized clinical trial found that the use of the cognitive aid tablet application improved adherence to resuscitation guidelines, thus demonstrating promise for improving patient outcomes, although further studies are necessary to confirm these findings and demonstrate its impact in clinical practice.

## Teaser

This randomized clinical trial assesses the effectiveness of a cognitive aid tablet application in improving the management of simulated in-hospital pediatric cardiac arrest.

## Introduction

Pediatric cardiac arrest is a rare emergency associated with high mortality and important clinical sequelae.^1,2^ Deviations from international resuscitation guidelines often occur and can negatively affect patient outcomes.^3,4,5^ Electronic and paper-based tools have been developed to support resuscitation teams in optimizing the management of cardiac arrest, but most of them are focused on adult cardiopulmonary resuscitation (CPR) in an out-of-hospital setting^6^ or on the quality of chest compressions through audio and/or visual feedback.^7^ Several electronic cognitive aids have been developed to improve adherence to resuscitation guidelines; however, most of these tools are not tested in terms of content, usability, and users’ perceived workload.^8^ To our knowledge, no real-event study^9^ and a limited number of simulation-based studies have tested the impact of cognitive aids on the management of cardiac arrest. Most of these studies focused on adult case scenarios, included a limited sample size, and presented important methodological limitations.^10^

In 2019, we developed an interactive, multimodal, electronic cognitive aid in the form of a tablet application (app), named PediAppRREST, to provide decision support to the team leader through a clickable list of actions to be performed in a stepwise manner, closely following the American Heart Association (AHA) resuscitation guidelines. The app was developed and refined through an iterative prototyping approach on the basis of users’ needs and feedback^11^ to overcome the most frequent deviations detected in simulation-based observational studies.^12,13,14^ The objective of this study was to test the effectiveness of the PediAppRREST app in reducing deviations from AHA resuscitation guidelines during the management of simulated pediatric cardiac arrest.

## Methods

This randomized clinical trial was deemed a negligible risk study by the human ethics committee of the University Hospital of Padua and granted approval via a fast-tracked review process. All participants provided written informed consent. The study was reported according to the extended Consolidated Standards of Reporting Trials (CONSORT Extended) reporting guidelines for health care simulation-based research. The trial protocol and statistical analysis plan are provided in Supplement 1.

### Design, Setting, and Participants

This was a multicenter, simulation-based, 3-group parallel randomized clinical trial. The study was carried out between September 2020 and December 2021 at 4 Italian University Hospitals (Padua, Florence, Rome, and Novara), with analysis performed between January and June 2022. Residents in pediatrics, emergency medicine, and anesthesiology were recruited after providing written informed consent. Adult and pediatric Basic and Advanced Life Support–certified residents, following the AHA^3^ or the European Resuscitation Council recommendations,^4^ were eligible. Only Pediatric Advanced Life Support (PALS)–certified participants were eligible for the team leader role. Exclusion criteria were participation in the previous pilot study of the PediAppRREST app,^11^ or personal leave during the study period.

### Interventions

Participants were stratified by hospital location and residency specialty. Among PALS-certified residents, 1 participant was randomly assigned to each team with the role of team leader. Among the remaining PALS- and non-PALS–certified residents, 2 participants were randomly assigned to each team to obtain teams of 3 members, with at least 1 PALS-certified resident for the team leader role. Teams were block randomized to 1 of 3 study groups in a 1:1:1 ratio using a computer-based sequence and sealed envelopes. All the teams conducted the same scenario of pediatric cardiac arrest using a different cognitive aid according to the randomization allocation: the PediAppRREST app (PediAppRREST intervention group); a paper-based cognitive aid, the AHA PALS pocket reference card (PALS control group); or no cognitive aid (null control group). The standard simulation was a 10-minute scenario of nonshockable in-hospital pediatric cardiac arrest^3^ conducted in an off-site setting (eAppendix 1 in Supplement 2). A simulation team member played the role of a nurse and acted according to a predetermined script.^15^ The manikin (Resusci Junior QCPR; Laerdal), the environment recreating an emergency department resuscitation room, and the study procedures were standardized at each study site. Further information about the study procedures and methods have been described in the trial protocol in Supplement 1 and elsewhere.^16^

### Outcomes

Two independent and trained reviewers (G.T. and E.F.)^17,18^ with clinical and simulation expertise assessed study outcomes by reviewing the video recordings of all the study scenarios. Disagreements were resolved by a third reviewer (G.M.). Interrater reliability between the 2 reviewers was measured. Participants and outcome assessors’ blinding was not possible due to the nature of the interventions.

#### Primary Outcome

The main outcome of the study was the number of deviations from PALS guidelines during the management of pediatric cardiac arrest, as measured by a 15-item checklist. For each item, a score of 1 point was assigned if a deviation (ie, delay, error, or omission) occurred, while zero was assigned if the action was correctly and timely performed. Scores ranged from 0 to 15, with lower the scores indicating fewer deviations from guidelines.^16^ Details of the checklist items are available in the Box. The checklist includes items from PALS recommendations,^3,4,19^ worded based on published checklists, scores, and tools.^5,20,21,22,23,24,25^ A 16-item version of the checklist was used as an outcome measure in the pilot study,^11^ following which the checklist was revised and finalized into the 15-item error score. This was used as a primary outcome measure in an observational study, and was shown to have good interrater reliability (concordance correlation coefficient [CCC], 0.887) between 2 trained video reviewers.^12^

Box. **Error Score Items for Nonshockable Pediatric Cardiac Arrest**

1. CPR started within 30 s from recognition of pulseless state.
2. CPR board or rigid surface positioned underneath the manikin within 60 s from recognition of pulseless state.
3. Compression and ventilation ratio of 15:2.
4. Help called (hospital emergency response system activated) within 60 s from recognition of pulseless state.
5. Compressors switched more than once during CPR.
6. EKG monitoring started within 60 s from recognition of pulseless state.
7. IV or IO access called within 60 s from recognition of pulseless state.
8. First epinephrine called within 30 s from recognition of pulseless state.
9. First epinephrine administered at the correct dose and dilution and by the correct route (IV or IO), followed by a normal saline flush, while compressions are being performed, within 180 s (3 min) from recognition of pulseless state.^a^
10. Second epinephrine called between 3 and 5 min from the first administration of epinephrine.
11. Second epinephrine administered at the correct dose and dilutiona and by the correct route, followed by a normal saline flush, while compressions are being performed, within 5 min from the first epinephrine.
12. Blood gas called during cardiac arrest.
13. Reversible causes treated.
14. Shock not administered.
15. Medications other than epinephrine (eg, amiodarone, lidocaine, atropine) not administered.^b^

Abbreviations: CPR, cardiopulmonary resuscitation; EKG, electrocardiogram; IO, intraosseous; IV, intravenous.

^a^
Correct dose of epinephrine is defined as 0.01 mg/kg (or a deviation from the correct weight dose of <10%); correct dilution of epinephrine is defined as 1:10.000 (0.1 mg/mL).

^b^
Administration of medications to treat identified reversible causes is not considered in this item.

#### Secondary Outcomes

We assessed 6 secondary outcomes. Performance and time (in seconds) to accomplish critical resuscitation interventions were measured.^3,16^ Chest compression quality metrics were analyzed by the manikin internal software (Skill Reporter, Laerdal). Chest compression quality was defined according to AHA standards as proportion of compressions with depth 50 to 60 mm, compression fraction (percentage of time during arrest with compressions), mean depth of compressions, and mean rate of compressions.^3,16,26^ Team clinical performance was evaluated by means of the asystole section of the Clinical Performance Tool (range, 0-13; higher score indicating better performance), a scoring system with supportive validity evidence designed on PALS algorithms.^20,21^ Usability of the app was evaluated by team leaders who used the app through a questionnaire that included open-ended questions and a score with published supportive validity evidence, the System Usability Scale (range, 0-100; higher score indicating better usability).^27,28^ Team leaders’ workload was measured by the raw version of the NASA-Task Load Index, which includes 6 subscale scores representing different perceived workload domains (range, 0-100; higher score indicating higher perceived workload).^29,30^

### Statistical Analysis

Sample size was calculated considering the results obtained during our previous observational^12^ and pilot^11^ simulation-based studies, in which we found a within-group SD of 2.2. Each study group needed 29 teams to identify a difference of at least 3.0 points in the error score (ie, 20% difference in the primary outcome measure) using the Tukey-Kramer (pairwise) multiple comparison procedure at a 5% significance level and 80% power (PASS sample size software version 11; NCSS). In consideration of possible technical issues, we planned to boost the sample size by 20% per group, aiming to include 35 teams in each group, with a total of 105 teams (315 residents).

The results were summarized with counts and percentages for categorical variables and mean and SD for normally distributed quantitative variables. For variables with asymmetric distribution, we also reported the median and IQR. The data were analyzed with generalized linear models considering the analysis of variance for quantitative variables, binomial distribution for binary data, generalized logit for multinomial nonordinal data, and cumulative logit for multinomial ordinal data (eAppendix 2 in Supplement 2). In case of statistical significance, pairwise comparisons were performed adjusting the *P* values and the 95% CIs for the primary outcome with the Tukey-Kramer method to take into account the multiplicity of the comparisons. *P* values were 2-sided, and *P* < .05 was considered indicative of statistical significance. Agreement between reviewers for error scores was calculated on primary outcome measures through the CCC and its 95% CI calculated with the bootstrap method considering 2000 resamplings.^31^ All the analyses were performed considering the intention-to-treat principle (participants analyzed as randomized); a per-protocol analysis (excluding participants with protocol deviation) was also performed for the primary outcome. The statistical analysis was performed with SAS statistical software version 9.4 (SAS Institute) for Windows.

## Results

We recruited 324 residents (108 teams). Eight teams did not participate in the trial due to sickness or withdrawal of consent, leaving 100 teams for the intention-to-treat analysis: 32 teams in the PediAppRREST group, 35 in the PALS group, and 33 in the null control group (Figure). Participant and team leader characteristics (mean [SD] age, 29.0 [2.2] years; 195 [65%] female; 210 residents [70%] in pediatrics, 48 residents [16%] anesthesiology; 42 residents [14%] emergency medicine) and previous experiences in simulation and management of real cardiac arrest events were comparable among the 3 groups (Table 1). Team distribution among study sites is available in eTable 1 in Supplement 2.

**Figure.**
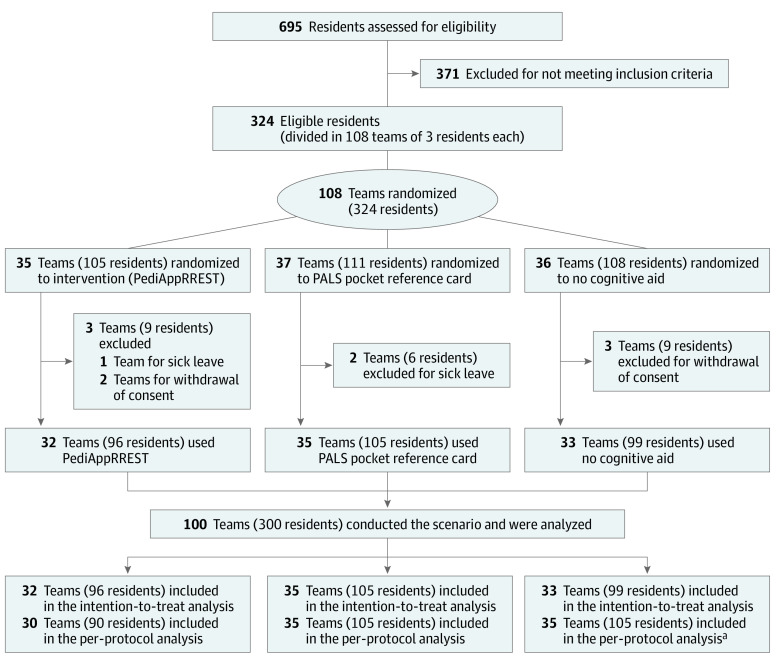
Study Recruitment Flowchart ^a^Two teams (6 residents) from the PediAppRREST group decided not to use the application.

**Table 1.** Participant Demographic Characteristics and Previous Experience in Simulation and Resuscitation

Characteristic	Participants by group, No. (%)		
	PediAppRREST (n = 96)	PALS (n = 105)	Null control (n = 99)
**All participants**			
Age, mean (SD), y	28.7 (1.9)	29.3 (2.6)	29.0 (2.0)
Residency program			
Pediatrics	66 (68.8)	75 (71.5)	68 (69.4)^a^
Anesthesiology	18 (18.8)	18 (17.1)	12 (12.2)^a^
Emergency medicine	12 (12.5)	12 (11.4)	18 (18.4)^a^
Year of residency program			
First	24 (25.0)	20 (19.0)	16 (16.2)
Second	33 (34.4)	37 (35.2)	36 (36.4)
Third	19 (19.8)	18 (17.1)	18 (18.2)
Fourth	7 (7.3)	15 (14.3)	12 (12.1)
Fifth	13 (13.5)	15 (14.3)	17 (17.2)
BLS certification	81 (84.4)	78 (75.0)^a^	72 (72.7)
Time since BLS certification, mo			
Mean (SD)	35.3 (25.7)	31.5 (21.4)	34.3 (24.0)
Median (IQR)	26.5 (17.0-48.0)^a^	25.0 (18.0-37.0)^b^	24.5 (15.0-56.0)
PBLS certification	66 (68.8)	69 (65.7)	66 (66.7)
Time since PBLS certification, mo			
Mean (SD)	21.7 (15.9)	21.1 (14.8)	26.8 (39.0)
Median (IQR)	18.0 (12.0-30.0)	18.0 (10.0-26.5)	18.5 (12.0-36.0)
ALS/ACLS certification	33 (34.4)	30 (29.4)^c^	29 (29.6)
Time since ALS/ACLS certification, mo			
Mean (SD)	19.5 (18.2)	19.2 (15.4)	21.7 (22.8)
Median (IQR)	17.0 (4.0-24.0)	18.0 (8.0-24.0)^a^	12.0 (2.0-36.0)^a^
PALS certification	33 (34.7)^a^	36 (34.3)	34 (34.7)^a^
Time since PALS certification, mo			
Mean (SD)	19.6 (20.0)	11.1 (8.9)	13.3 (13.1)
Median (IQR)	12.0 (2.5-31.0)^a^	11.5 (3.0-18.0)^b^	11.0 (2.0-20.0)
Participated in high fidelity simulation	67 (70.5)^a^	66 (63.5)^a^	61 (61.6)
Simulations conducted during the last year, No			
0	33 (34.4)	48 (45.7)	42 (42.4)
1-5	54 (56.3)	50 (47.6)	51 (51.5)
6-10	7 (7.3)	5 (4.8)	3 (3.0)
>10	2 (2.1)	2 (1.9)	3 (3.0)
Time since last simulation, mo			
Mean (SD)	11.8 (13.5)	8.3 (8.4)	9.2 (9.9)
Median (IQR)	8.0 (2.0-15.0)^b^	7.0 (2.0-12.0)^a^	6.0 (1.0-12.0)
Pediatric simulation	57 (59.4)	55 (53.4)^b^	52 (52.5)
CPR simulation	62 (64.6)	67 (63.8)	67 (67.7)
Time since last CPR simulation, mo			
Mean (SD)	13.6 (16.2)	10.2 (9.0)	11.5 (12.3)
Median (IQR)	7.5 (2.0-18.0)	9.0 (2.0-13.0)	8.0 (2.0-18.0)
CPR simulations during last year, mean (SD), No.	1.9 (0.7)	1.8 (0.7)	1.8 (0.6)
Experience of real CPR events	37 (38.5)	48 (45.7)	49 (49.5)
Real CPR events experienced, No.			
Mean (SD)	13.2 (24.3)	5.8 (9.1)	7.3 (12.8)
Median (IQR)	3.0 (1.0-10.0)	3.0 (1.0-6.0)	3.0 (1.0-8.0)
Time since the last experienced real CPR event, mo			
Mean (SD)	13.3 (16.0)	14.3 (15.2)	16.7 (23.1)
Median (IQR)	5.0 (2.0-24.0)	8.0 (2.5-23.0)	6.0 (2.0-24.0)
Real CPR events witnessed during the last year, No.			
Mean (SD)	3.8 (6.8)	2.9 (7.1)	2.4 (3.1)
Median (IQR)	1.0 (0.0-5.0)	1.0 (0.0-3.0)	1.0 (0.0-4.0)
**Analysis limited to team leaders only** ^d^			
Age, mean (SD), y	30.0 (1.8)	31.1 (3.0)	30.8 (2.1)
Residency program			
Pediatrics	22 (68.8)	25 (71.4)	23 (69.7)
Anesthesiology	6 (18.8)	6 (17.1)	4 (12.1)
Emergency medicine	4 (12.5)	4 (11.4)	6 (18.2)
Year of residency program			
First	2 (6.3)	0	1 (3.0)
Second	2 (6.3)	3 (8.6)	2 (6.1)
Third	9 (28.1)	7 (20.0)	7 (21.2)
Fourth	6 (18.8)	10 (28.6)	8 (24.2)
Fifth	13 (40.6)	15 (42.9)	15 (45.5)
PALS certification	32 (100)	35 (100)	33 (100)
Time since PALS certification, mo			
Mean (SD)	19.4 (20.3)	10.9 (8.9)	13.6 (13.1)
Median (IQR)	12.0 (2.0-32.0)^a^	11.0 (3.0-18.0)^b^	11.0 (2.0-20.0)
Participated in high fidelity simulation	31 (96.9)	33 (94.3)	30 (90.9)
Simulations conducted during the last year, No			
0	6 (18.8)	7 (20.0)	6 (18.2)
1-5	21 (65.6)	25 (71.4)	21 (63.6)
6-10	3 (9.4)	1 (2.9)	3 (9.1)
>10	2 (6.3)	2 (5.7)	3 (9.1)
Time since last simulation, mo			
Mean (SD)	12.5 (16.8)	7.8 (6.6)	9.7 (9.7)
Median (IQR)	7.0 (1.0-12.0)	7.0 (2.0-12.0)	7.5 (1.0-15.0)
Pediatric simulation	29 (90.6)	28 (80.0)	24 (72.7)
CPR simulation	31 (96.9)	32 (91.4)	33 (100)
Time since last CPR simulation, mo			
Mean (SD)	13.5 (17.4)	9.0 (6.9)	11.0 (9.6)
Median (IQR)	7.0 (2.0-18.0)	9.5 (2.0-12.5)	8.0 (2.0-18.0)
CPR simulations during last year, No. (%)			
0	7 (22.6)	10 (31.3)	11 (33.3)
1-5	21 (67.7)	19 (59.4)	17 (51.5)
6-10	0	1 (3.1)	5 (15.2)
>10	3 (9.7)	2 (6.3)	0
Experience of real CPR events	18 (56.3)	18 (51.4)	22 (66.7)
Real CPR events experienced, No.			
Mean (SD)	22.2 (32.5)	7.3 (11.6)	10.1 (17.8)
Median (IQR)	4.0 (2.0-30.0)	3.5 (1.0-10.0)	2.5 (1.0-15.0)
Time since the last experienced real CPR event, mo			
Mean (SD)	14.9 (19.2)	10.7 (13.3)	13.0 (18.4)
Median (IQR)	5.5 (1.0-24.0)	6.0 (1.0-12.0)	4.5 (1.0-18.0)
Real CPR events witnessed during the last year, No.			
Mean (SD)	5.7 (9.1)	5.2 (11.2)	2.8 (3.9)
Median (IQR)	1.0 (0.0-5.0)	1.0 (0.0-5.0)	1.0 (0.0-6.0)

Abbreviations: ALS, Advanced Life Support; ACLS, Advanced Cardiac Life Support; BLS, Basic Life Support; CPR, cardiopulmonary resuscitation; PALS, Pediatric Advanced Life Support; PBLS, Pediatric Basic Life Support.

^a^ Data missing for 1 participant.

^b^ Data missing for 2 participants.

^c^ Data missing for 3 participants.

^d^ Includes data for 32 team leaders in the PediAppRREST group, 35 team leaders in the PALS control group, and 33 team leaders in the null control group.

### Primary Outcome

The mean number of deviations from guideline recommendations, as measured by the error score, was significantly lower in the PediAppRREST group (mean [SD] 3.4 [2.0] points) compared with the 2 control groups (PALS: mean difference, −3.0; 95% CI, −4.0 to −1.94; *P* < .001; null control: mean difference, −2.6; 95% CI, −3.6 to −1.5; *P* < .001) (Table 2). No statistically significant difference in error scores was found between control groups (mean difference, 0.4; 95% CI, −0.6 to 1.5; *P* = .59). Analyzing the individual items of the score, significant differences in the intervention group compared with the control groups were detected for CPR board positioning, call for help, first epinephrine administration, and second epinephrine administration (Table 2). The interrater reliability for error scoring was substantial^32^ (CCC, 0.960; 95% CI, 0.925 to 0.976).

**Table 2.** Error Scores Overall and for Individual Items

Item	No. (%)			*P* value				Tukey-Kramer adjusted pairwise comparisons, rate difference (95% CI)		
	PediAppRREST group (n = 32)	PALS group (n = 35)	Null control group (n = 33)	Overall	Tukey-Kramer adjusted pairwise comparisons					
					PediAppRREST vs PALS	PediAppRREST vs null control	PALS vs null control	PediAppRREST vs PALS	PediAppRREST vs null control	PALS vs null control
Overall score, mean (SD)^a^	3.4 (2.0)	6.4 (1.8)	6.0 (1.6)	<.001	<.001	<.001	.59	−3.0 (−4.0 to −1.9)^b^	−2.6 (−3.6 to −1.5)^b^	0.4 (−0.6 to 1.5)^b^
Individual score items^c^										
CPR started within 30 s	26 (81.3)	30 (85.7)	29 (87.9)	.76	.88	.74	.96	−4.5 (−26.1 to 17.2)	−6.6 (−27.9 to 14.7)	−2.2 (−21.7 to 17.4)
Position CPR board	22 (68.8)	6 (17.1)	6 (18.2)	<.001	<.001	<.001	.99	51.6 (26.9 to 76.3)	50.6 (25.4 to 75.8)	−1.0 (−23.1 to 21.0)
Correct compression to ventilation ratio	31 (96.9)	33 (94.3)	28 (84.8)	.23	.86	.20	.41	2.6 (−9.3 to 14.5)	12.0 (−4.5 to 28.6)	9.4 (−8.1 to 27.0)
Call for emergency team	20 (62.5)	1 (2.9)	1 (3.0)	<.001	<.001	<.001	.10	59.6 (38.2 to 81.1)	59.5 (37.9 to 81.0)	−0.2 (−9.9 to 9.6)
Compressors switched	25 (78.1)	14 (40.0)	22 (66.7)	.003	.002	.55	.06	38.1 (11.8 to 64.4)	11.5 (−14.7 to 37.6)	−26.7 (−54.4 to 1.1)
EKG-monitoring	30 (93.8)	29 (82.9)	31 (93.9)	.30	.34	1.00	.32	10.9 (−7.4 to 29.2)	−0.2 (−14.4 to 14.0)	−11.1 (−29.2 to 7.2)
IV/IO access	16 (50.0)	20 (57.1)	20 (60.6)	.68	.83	.66	.95	−7.1 (−36.1 to 21.8)	−10.6 (−39.8 to 18.6)	−3.5 (−31.9 to 24.9)
First epinephrine called within 30 s	1 (3.1)	4 (11.4)	1 (3.0)	.36	.38	1.00	.36	−8.3 (−23.1 to 6.4)	0.1 (−10.1 to 10.3)	8.4 (−6.2 to 23.0)
First epinephrine administered correctly^d^	27 (84.4)	12 (34.3)	7 (21.2)	<.001	<.001	<.001	.45	50.1 (25.6 to 74.6)	63.2 (40.4 to 86.0)	13.1 (−12.5 to 38.6)
Second epinephrine called	25 (78.1)	17 (48.6)	20 (60.6)	.03	.03	.27	.58	29.6 (3.0 to 56.1)	17.5 (−9.2 to 44.2)	−12.0 (−40.6 to 16.5)
Second epinephrine administered correctly^d^	26 (81.3)	9 (25.7)	8 (24.2)	<.001	<.001	<.001	.99	55.5 (31.5 to 79.6)	57.0 (32.8 to 81.2)	1.5 (−23.5 to 26.5)
Call for blood gas	30 (93.8)	27 (77.1)	28 (84.8)	.12	.12	.47	.69	16.6 (−3.1 to 36.3)	8.9 (−9.1 to 26.9)	−7.7 (30.2 to 14.8)
Treatment of reversible causes	30 (93.8)	30 (85.7)	32 (97.0)	.24	.52	.81	.21	8.0 (−9.3 to 25.4)	−3.2 (−15.6 to 9.2)	−11.3 (−27.0 to 4.5)
Defibrillation not performed^e^	31 (96.9)	34 (97.1)	33 (100)	.46	1.00	.73	NE	−0.3 (−10.2 to 9.7)	−3.2 (−13.1 to 6.8)	−29 (NE)
Medications other than epinephrine not administered^f^	31 (96.9)	35 (100)	32 (97.0)	.98	.75	>.99	NE	−3.1 (−13.3 to 7.1)	−0.1 (−10.3 to 10.1)	3.3 (NE)

Abbreviations: CPR, cardiopulmonary resuscitation; EKG, electrocardiogram; IO, intraosseous; IV, intravenous; NE, not estimable; PALS, Pediatric Advanced Life Support.

^a^ Range, 0 to 15, with higher score indicating more errors.

^b^ Expressed as mean difference (95% CI).

^c^ Details of the correct performance of each item are provided in the Box.

^d^ Correct dose of epinephrine is defined as 0.01 mg/kg (or a deviation from the correct weight dose of <10%); correct dilution of epinephrine is defined as 0.1 mg/mL (1:10.000).

^e^ The defibrillation is not indicated in the PALS algorithm for nonshockable rhythm.

^f^ Administration of medications to treat identified reversible causes is not considered in this item.

Analyses stratified by residency programs showed that in each residency group, the mean number of deviations from guidelines was lower in the PediAppRREST group than in the control groups (eTable 2 in Supplement 2). The per-protocol analysis showed similar results to the intention-to-treat analysis. The mean number of deviations was significantly lower in the PediAppRREST group (mean [SD] score, 3.4 [2.0] points) compared with the PALS group (mean difference, −3.0; 95% CI, −4.1 to −1.9; *P* < .001) and null control (mean difference, −2.6; 95% CI, −3.7 to −1.5; *P* < .001) groups. The mean number of deviations in the 2 control groups did not significantly differ (mean difference, 0.4; 95% CI, −0.6 to −1.5; *P* = .59).

### Secondary Outcomes

Use of the PediAppRREST app, compared with both control groups, was associated with a significantly higher percentage of teams that positioned a CPR board or rigid surface underneath the patient (78.1% of PediAppRREST teams; 20.0% of PALS teams; *P* < .001; 27.3% of null control teams; *P* < .001), correctly administered saline flush after epinephrine (87.5% of PediAppRREST teams; 34.3% of PALS teams; *P* < .001; 27.3% of null control teams; *P* < .001), and achieved return of spontaneous circulation (ROSC; 78.1% of PediAppRREST teams; 28.6% of PALS teams; *P* < .001; 18.2% of null control teams; *P* < .001) (eTable 3 in Supplement 2). The analysis of the time to perform critical resuscitation actions showed that teams that used the PediAppRREST app took significantly shorter times in calling for help (median [IQR], 88.5 [56.0 to 107.0] seconds) compared with the PALS group (median [IQR], 275.5 [192.5 to 350.5] seconds; *P* < .001) and the null control group (median [IQR], 272.5 [204.0 to 388.5] seconds; *P* < .001) (eTable 4 in Supplement 2).

The analysis of chest compression quality metrics did not show any statistically significant difference between the 3 study groups (Table 3). The clinical performance of the teams measured by the Clinical Performance Tool score, was significantly higher in the PediAppRREST group (mean [SD], 8.9 [1.6]) than in the PALS group (mean difference, 1.4; 95% CI, 0.4 to 2.3; *P* = .002), and null control group (mean difference, 1.1; 95% CI, 0.2 to 2.1; *P* = .01) group (Table 3; eTable 5 in Supplement 2).

**Table 3.** Principle Secondary Outcomes Results

Item	PediAppRREST group (n = 29)^a^	PALS group (n = 29)^a^	Null control group (n = 28)^a^	*P* value				Tukey-Kramer adjusted pairwise comparisons, mean difference (95% CI)		
				Overall	Tukey-Kramer adjusted pairwise comparisons			PediAppRREST vs PALS	PediAppRREST vs null control	PALS vs null control
					PediAppRREST vs PALS	PediAppRREST vs null control	PALS vs null control			
CC quality metrics										
CC depth, mean (SD), mm	46.3 (6.1)	44.8 (6.7)	47.3 (6.0)	.32	.61	.84	.30	1.6 (−2.4 to 5.5)	−0.9 (−4.9 to 3.0)	−2.5 (−6.5 to 1.5)
Chest recoil, % of CCs										
Mean (SD)	72.2 (26.9)	82.5 (22.0)	71.3 (20.9)	.14	.22	.99	.18	−10.3 (−25.0 to 4.4)	0.9 (13.9 to 15.8)	11.2 (−3.6 to 26.0)
Median (IQR)	83.0 (53.0 to 94.0)	92.0 (75.0 to 97.0)	75.0 (52.0 to 87.0)							
Proportion of CC with depth 50-60 mm, %										
Mean (SD)	40.1 (31.6)	33.8 (31.8)	43.5 (30.8)	.49	.73	.91	.47	6.3 (−3.4 to 26.0)	−3.5 (−23.3 to 16.4)	−9.8 (−29.6 to 10.1)
Median (IQR)	33.0 (17.0 to 69.0)	23.0 (8.0 to 61.0)	43.5 (14.0 to 66.5)							
Mean CC rate, mean (SD), min^−1^	108.0 (17.9)	112.5 (11.7)	112.3 (13.0)	.42	.47	.51	>.99	−4.5 (13.5 to 4.6)	−4.3 (−13.4 to 4.9)	0.2 (−8.9 to 9.4)
CC fraction, %										
Mean (SD)	66.9 (15.7)	64.2 (20.7)	66.8 (21.0)	.84	.86	>.99	.87	2.7 (−9.4 to 14.8)	0.1 (−12.0 to 12.3)	−2.5 (−14.7 to 9.6)
Median (IQR)	67.0 (60.0 to 74.0)	64.0 (60.0 to 77.0)	74.0 (63.5 to 79.0)							
CPT total score (0-13), mean (SD)^a^	8.9 (1.6)	7.5 (1.5)	7.7 (1.6)	.001	.002	.01	.84	1.4 (0.4 to 2.3)	1.1 (0.21 to 2.1)	−0.1 (−1.2 to 0.7)
Total R-TLX score, mean (SD)^b^	58.1 (13.9)	60.2 (12.5)	61.3 (12.1)	.60	.79	.58	.94	−2.1 (−9.6 to 5.5)	−3.2 (−10.7 to 4.4)	−1.1 (−8.6 to 6.4)

Abbreviations: CC, chest compression; CPT, Clinical Performance Tool; PALS, Pediatric Advanced Life Support; R-TLX, raw NASA Task Load Index.

^a^ Scores range from 0 to 13, with higher score indicating better performance. Includes data for 32 PediAppRREST teams, 35 PALS teams, and 33 null control teams.

^b^ Scores range from 0 to 100, with higher score indicating higher perceived workload. Includes data for 32 PediAppRREST teams, 34 PALS teams, and 33 null control teams.

The System Usability Scale scores showed a mean (SD) score of 74.8 (16.1) points. This result corresponds to a good rating (similar to a 5 on a 1-7 Likert scale).^33^ The users’ most frequent issues and suggestions to improve the app were the possibility that its use could distract from getting the overview of management actions and the need of some users to visualize the entire resuscitation algorithm (eTable 6 in Supplement 2). Strategies to improve the app and its use were identified based on users’ suggestions (eTable 6 in Supplement 2).

Team leaders’ workload was not statistically different among the 3 study groups. However, by analyzing the individual items of the score, the mean values for the mental demand item was significantly lower in the intervention group (mean [SD] score, 70.0 [20.9] points) than in the PALS (mean [SD] score, 81.3 [14.7]; *P* = .02) and null control (mean [SD] score, 80.5 [13.4]; *P* = .03) groups (Table 3; eTable 7 in Supplement 2).

## Discussion

In this randomized clinical trial, we analyzed the effect of the use of the PediAppRREST tablet app on the management of a simulated case of pediatric cardiac arrest and found that its use was associated with fewer deviations from guidelines and a better team clinical performance compared with the use of the AHA reference pocket card and no cognitive aid. Given that deviations from recommendations are still frequent and are proven to affect clinical outcomes,^5^ our findings highlight the potential of our interactive multimodal tablet app to improve the management of pediatric cardiac arrest and hence contribute to increased survival of children experiencing cardiac arrest.

The detected benefits of using the PediAppRREST app in our study could be related to its ability to provide multimodal interactive stepwise decision support to the team leader. In contrast, paper-based aids are static and usually display the entire algorithms in a 1-page format, requiring users to independently identify the correct steps in the algorithm and rapidly move through its decision nodes and treatment recommendations. This process can cause a mental overload in the users and in turn delay actions and increase management errors. Given the low complexity of the PediAppRREST cognitive aid, needing only a tablet as hardware support, it could potentially be easily implemented also in lower-resource settings. In addition, practice with the app through mental simulation could be facilitated and promoted by its download on personal smartphones.

A 2022 meta-analysis^10^ that included mostly adult cardiac arrest scenarios and 2 pediatric studies^34,35^ reported that the use of any cognitive aid (electronic or paper-based) was associated with a better team performance compared with teams that did not use any cognitive aid. However, the use of an electronic cognitive aid was associated with a better team performance compared with a paper-based cognitive support.^10^ These findings partly align with our study results, which showed a better resuscitation performance in teams using our electronic cognitive aid, but no difference between teams that used the paper-based aid and those using no cognitive aid. This divergence could be explained by differences in the cognitive supports tested, study designs, outcome measures, and scenarios.

To our knowledge, only 1 other study has compared the effect of an electronic (smartphone app) cognitive aid with a paper-based aid and no cognitive aid, and it was conducted using adult case scenarios.^36^ Although different from the cognitive tool we developed, Donzé et al^36^ similarly described a significantly better performance in the teams that used the electronic cognitive aid compared with the control groups. In addition, the comparison between the 2 control groups showed no significant differences in the management of the cardiac arrest scenario,^36^ as reported in our study.

With respect to the quality of CPR, we found that the use of the app did not impact chest compression quality metrics. However, this cognitive tool was not developed to support the compressors during CPR, thus an improvement of the quality of compressions was not expected. Different real-time feedback devices and a CPR coach have been proven to effectively improve the quality of compressions.^3,7,26,37^ These strategies could be easily integrated with the use of PediAppRREST to further optimize the management of cardiac arrest.

Overall, our study found that the use of the PediAppRREST app globally improved the management of pediatric cardiac arrest, mostly by reducing omissions and errors that can impact clinical outcomes. The use of our app was associated with a higher proportion of ROSC than control groups. However, in our study, ROSC was achieved when predefined tasks were correctly completed during the management of the scenario. For this reason, while ROSC is an important outcome in real-life studies, it was not included as an outcome in our simulation-based trial.^10^

Most of the times to perform critical actions for resuscitation did not significantly differ between the study groups. This could be related to the limited prior exposure to the app by the team leaders, as the use of a new cognitive tool, by itself, takes time and cognitive effort. Professionals who frequently lead pediatric clinical emergencies and are not completely familiar with a cognitive aid could choose not to use it or could be delayed in their management by the tool; in contrast, cognitive aids could be more beneficial for less expert clinicians, for both ensuring all tasks are completed and for expediting management goals. Future studies should ascertain whether by improving users’ proficiency and efficiency in navigating PediAppRREST via increased practice could result in a reduction in time to perform critical actions compared with standard practice.

While any cognitive support could potentially increase users’ workload, as it may distract from the management of the scenario by requiring extra attention and concentration to understand its use or follow its guidance, we found that the use of PediAppRREST was not associated with a higher perceived workload among users, and its usability was rated as good.^33^ The novel cognitive aid was also reported to have a lower mental demand compared with both control groups, similar to other studies of cognitive aids.^10,33,34,37,38^ This could be related to its ability to ease the cognitive burden of some tasks (eg, calculating drug doses, follow the correct sequence and timing of resuscitation actions, remembering the possible reversible causes and their treatments). Our study also offered an opportunity to collect participants’ issues and suggestions, which were then used to optimize the app (ie, by adding the flowcharts in a section of the app) and its use (ie, by choosing a dedicated team member to use the app, different from the team leader), through a continuous iterative process encouraging personal users’ preferences, with the purpose of individualize the use of the app and improve its usability.

### Limitations

This study has some limitations. The main limitation is that only residents were included as participants. We elected to include residents because they are frequently the frontline physicians managing the first minutes of a pediatric cardiac arrest in many institutions.^10,34,35,39,40,41,42,43,44^ In our study, resuscitation teams were comprised of only 4 members (3 residents and 1 actor playing the nurse role); had we recruited a larger team, including attending physicians and physicians from different settings, this may have potentially influenced our outcomes and the usability ratings of the app. The second limitation is that the error score used to assess the primary outcome has not been extensively validated. However, none of the available validated tools was comprehensive enough to include all the deviations from guideline recommendations that were identified during previous observational studies^12,13,14^ and that could impact on clinical outcomes.^5,45^ A comprehensive tool was needed to accurately assess the possible impacts of the use of the PediAppRREST on the management of pediatric cardiac arrest. In addition, in October 2020, the AHA guidelines were updated^3^; thus, we verified that the content of PediAppRREST, and outcome measures were consistent with the new guideline recommendations.^3^ The paper-based cognitive aid used by the PALS control group was changed from the AHA 2015 PALS pocket reference card to the new version of the tool released in October 2020. Due to the nature of the intervention, participants and outcome assessors’ blinding was not possible, and this could have affected results, although we ensured blinding of the study statistician. Additionally, this is a simulation-based rather than a real-life study, which would be extremely challenging and ethically not justifiable to conduct in the emergency department setting due to the low frequency, unpredictability, and complexity of pediatric cardiac arrest events. Thus, caution is warranted before our results could be extended to clinical practice.

## Conclusions

In this randomized clinical trial, teams using the PediAppRREST app had fewer deviations from international guidelines and a better clinical team performance during the management of simulated pediatric cardiac arrest. Further studies are necessary to understand the effectiveness of this aid in multidisciplinary team compositions reflecting the latest PALS recommendations. Before considering implementation in clinical practice, systematic simulation training with the app and further studies should be performed.

## Notes

Supplement 1.
Trial Protocol and Statistical Analysis Plan

Supplement 2. **eAppendix 1.** Simulation Scenario **eAppendix 2.** Further Details Regarding Statistical Analysis **eTable 1.** Teams’ Distributions Between Study Site and Residency Program **eTable 2.** Subanalysis of Error Score by Residency Program **eTable 3.** Performance of Single Critical Resuscitation Actions **eTable 4.** Time to Perform Single Critical Resuscitation Actions **eTable 5.** Single Items of Clinical Performance Tool **eTable 6.** Issues in Using the PediAppRREST App and Proposed Solutions **eTable 7.** Single Items of Raw NASA Task Load Index

Supplement 3.
Data Sharing Statement
