# Supplementary material for: Effectiveness of a Novel Tablet Application in Reducing Guideline Deviations During Pediatric Cardiac Arrest: A Randomized Clinical Trial
Source: JAMA Netw Open. 2023 Aug 3;6(8):e2327272. doi: 10.1001/jamanetworkopen.2023.27272 (PMC10401301; doi:10.1001/jamanetworkopen.2023.27272)
Supplement: Supplement 1. — Trial Protocol and Statistical Analysis Plan [file jamanetwopen-e2327272-s001.pdf]

PediAppRREST - Effectiveness of a novel tablet app in reducing deviations from guidelines in the management of paediatric cardiac arrest

Supplementary file 1. PediAppRREST - Effectiveness of an interactive cognitive support tablet app in reducing deviations from guidelines in the management of paediatric cardiac arrest: protocol for a simulation-based randomized controlled trial

Replica of the protocol paper Corazza F et al, BMJ Open 2021

Authors:

Corazza F[1], Arpone M[1], Snijders D[1], Cheng A[2], Stritoni V[3], Ingrassia PL[4], De Luca M[5], Tortorolo L[6], Frigo AC[7], Da Dalt L[1], Bressan S[1].

Affiliations:

[1] Division of Paediatric Emergency Medicine, Department of Women's and Children's Health, University di Padua, Padua, Italy
[2]Departments of Paediatrics and Emergency Medicine, Alberta Children's Hospital, University of Calgary, Calgary, Canada
[3]Paediatric Intensive Care Unit, Department of Women's and Children's Health, University of Padua, Padua, Italy
[4]Interdepartmental Centre for Innovative Didactics and Simulation in Medicine and Health Professions, SIMNOVA, University of Piemonte Orientale, Novara, Italy
[5]Paediatric Emergency Medicine, Meyer University Hospital, University of Florence, Florence, Italy
[6]Institute of Intensive Care Medicine and Anaesthesiology, Agostino Gemelli University Hospital, Catholic University of the Sacred Heart, Rome, Italy
[7]Biostatistics, Epidemiology and Public Health Unit, Department of Cardiac, Thoracic, Vascular Sciences and Public Health, University of Padua, Padua, Italy.

SUMMARY

1. ABSTRACT
2. INTRODUCTION
    2.1 Objectives
3. METHODS AND ANALYSIS
    3.1 Study design and settings
    3.2 Participants
    3.3 Randomization, allocation concealment, and blinding
    3.4 Outcomes
        3.4.1 Primary outcome
        3.4.2 Secondary outcomes
    3.5 Intervention
        3.5.1 Intervention arm - PediAppRREST app
        3.5.2 Control arm - PALS pocket reference card
        3.5.3 Control arm - no cognitive aid
    3.6 Study procedures
    3.7 Data Collection and management
    3.8 Statistical Analysis
        3.8.1 Sample size calculation
        3.8.2 Data analysis plan
    3.9 Patient and Public Involvement
4. ETHICS AND DISSEMINATION
5. AUTHORS' CONTRIBUTIONS
6. FUNDING STATEMENT
7. COMPETING INTERESTS STATEMENT
8. REFERENCES
9. APPENDIX 1

1. ABSTRACT

Introduction: Paediatric cardiac arrest (PCA), despite its low incidence, has a high mortality. Its management is complex and deviations from guideline recommendations occur frequently. We developed a new interactive tablet app, named PediAppRREST, to support the management of PCA. The app received a good usability evaluation in a previous pilot trial. The aim of the study is to evaluate the effectiveness of the PediAppRREST app in reducing deviations from guideline recommendations in PCA management.

Methods and analysis: This is a multicentre, simulation-based, randomized controlled, three-parallel-arm study. Participants are residents in Paediatric, Emergency Medicine, and Anaesthesiology programs in Italy. All 105 teams (315 participants) manage the same scenario of in-hospital PCA. Teams are randomized by the study statistician into one of three study arms for the management of the PCA scenario: 1) an intervention group using the PediAppRREST app; or 2) a control group (CtrlPALS+) using the Paediatric Advanced Life Support (PALS) pocket reference card; or 3) a control group (CtrlPALS-) not allowed to use any PALS-related cognitive aid. The primary outcome of the study is the number of deviations (delays and errors) in PCA management from PALS guideline recommendations, according to a novel checklist, named c-DEV15plus. The c-DEV15plus scores will be compared between groups with a one-way ANOVA model, followed by the Tukey-Kramer multiple comparisons adjustment procedure in case of statistical significance.

Ethics and dissemination: The Ethics Committee of the University Hospital of Padova, coordinating centre of the trial, deemed the project to be a negligible risk study and approved it through an expedited review process. The results of the study will be disseminated in peer-reviewed journals, and at national and international scientific conferences. Based on the study results, the PediAppRREST app will be further refined and will be available for download by institutions/health care professionals.

Trial registration number: NCT04619498

2. INTRODUCTION

Paediatric cardiac arrest (PCA), despite its low incidence, is associated with high mortality and serious clinical sequelae.[1-5] The need for multiple rapid and complex interventions and the etio-pathogenic differences with adult cardiac arrest, make its management challenging and error prone. International scientific societies periodically release and update evidence-based guidelines outlining the recommended management of in-hospital and out-of-hospital PCA.[6-11] The Paediatric Advanced Life Support (PALS) course has been created by the American Heart Association (AHA) to train healthcare professionals in the advanced management of PCA.[12] Nevertheless, studies demonstrated that, despite training, deviations from guideline recommendations often occur in PCA management,[13-19] and lead to patients' worse clinical outcomes.[20,21]

Previous studies have assessed multiple strategies and tools to cognitively support providers to deliver optimal resuscitation during CA, as per guideline recommendations, showing variable efficacy.[22-36] Most of these studies focused on adult cardiopulmonary resuscitation (CPR) in an out-of-hospital setting and assessed pre-recorded audio/video support or contact by phone with a medical dispatcher.[32] Several studies have so far focused on technology developed to improve the quality of chest compressions through audio/visual feedback.[33-36] Software and apps for mobile phones, and tablets, as well as augmented reality glasses have been developed and used to improve adherence to guidelines.[23-31] However, most of these tools are directed to adult CA and showed to be associated with only partial improvements in the management of simulated CA scenarios. In addition, their usability and associated perceived workload has not been formally assessed before testing their efficacy. Overall, there is very limited experience on the usefulness of interactive cognitive support through an app in the management of PCA.[31]

We developed and refined a new audio-visual interactive app for tablets, named PediAppRREST, to support the management of PCA.[37] The app was developed based on the results of a multicentre observational simulation-based study, conducted by our research team, evaluating errors and delays in the management of a PCA scenario by paediatric residents.[38] We also tested the app usability in a pilot simulation-based study of non-shockable PCA scenarios, managed by paediatric residents, all PALS certified providers. The app received good usability evaluations and it did not increase the team leader's perceived workload compared to the control group that did not use any cognitive support tool.[37] No app that has previously been tested for usability and associated perceived workload, has so far been evaluated in a large,

appropriately powered, randomized clinical trial, involving physicians from different medical specialties, for its efficacy in guiding the management of PCA.

2.1 Objectives

The primary objective of this study is to determine, in a multicentre, randomized controlled trial, whether the use of the PediAppRREST app is associated with a reduction of deviations from international guidelines, in the management of a simulated PCA scenario, compared with the use of the PALS pocket reference card or with the use of no cognitive aid.

The secondary objectives are to further evaluate the usability of the PediAppRREST app and its impact on team members' workload, CPR quality, time to performance of critical resuscitation interventions, and overall team performance.

3. METHODS AND ANALYSIS

3.1 Study design and settings

The study is a national, multicentre, superiority, 3-parallel-group, randomized-controlled trial conducted in the setting of off-site intermediate-fidelity simulation. The study has been designed following guidelines for health care simulation research.[39] The study includes an intervention arm (PediAppRREST arm) and two control arms (CtrlPocketPALS+ and CtrlPocketPALS-). In the intervention arm participants use the novel interactive cognitive support tool, the PediAppRREST tablet app, to manage a standardized simulated scenario of PCA while the teams in the control arms manage the same scenario without the support of the app. Participants in the CtrlPocketPALS+ arm use the current recommended cognitive support tool, the PALS pocket reference card, while in the CtrlPocketPALS- arm no cognitive aid is used. The study design diagram is available in Figure 1. All the scenarios are video-recorded and reviewed by two previously trained and independent reviewers who will collect the data in the study Case Report Forms (CRFs).

3.2 Participants

Participants are recruited from medical residency programs in Paediatrics, Emergency Medicine and Anaesthesiology, at the i) University Hospital of Padua, University of Padua (Padua); ii) Meyer University Hospital, University of Florence (Florence); iii) Maggiore della Carità University Hospital, University of Piemonte Orientale (Novara); and iv) Agostino Gemelli University Hospital, Catholic University of Sacred Heart (Rome).

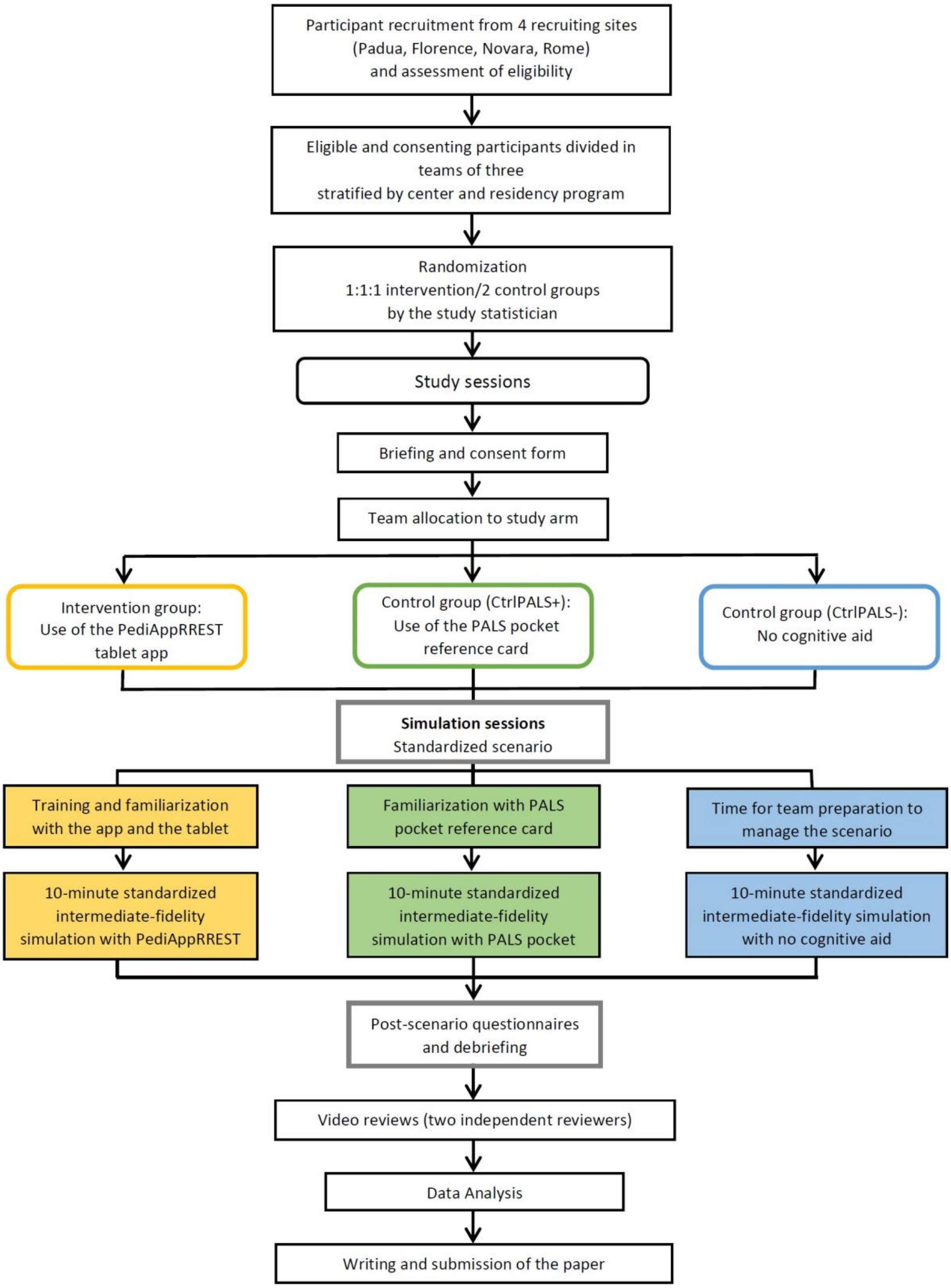

Figure 1. Study design diagram

Eligibility of potential participants is assessed by the study investigators of each participating site. Residents must meet all the following criteria to be enrolled in this trial: i) to be attending a residency training program in Paediatrics, Anaesthesiology or Emergency Medicine; ii) to be BLS (Basic Life Support) or P-BLS (Paediatric-Basic Life Support) or PALS or ALS (Advanced Life Support) or ACLS (Advanced Cardiac Life Support) certified, following the AHA or the European Resuscitation Council (ERC) recommendations; and iii) to give consent to participate to the study and to be video-recorded. Additionally, to be eligible for the role of team leader residents must be PALS-certified according to AHA or ERC guidelines. Residents who took part in the pilot study of the PediAppRREST app,[37] or who are unable to attend the simulation sessions because of maternity/paternity leave, sick/personal leave or training abroad are not eligible to participate in the trial.

3.3 Randomization, allocation concealment, and blinding

Participants are randomized by the study statistician in teams of three, stratified by study site and residency program specialty. Randomization is conducted assuring that in every team there will be at least one PALS-certified team member assigned to the role of team leader. The same statistician assigns a unique team identification number to each team and prepares the list of participants associated with each team to be sent to the principal investigators. Participants are randomly divided in teams of three members per each scenario; the team leader is selected among PALS-certified residents whereas the other two members have to be at least BLS-certified. The teams are randomized with a 1:1:1 ratio in the intervention arm (PediAppRREST arm) and the two control arms.

The statistician also creates opaque sealed envelopes externally marked with the team identification number and containing a paper slip, which indicates the arm allocation for that specific team. Team group allocation is concealed until the simulation sessions. Randomization of participants to teams, and teams to the arms, is performed with SAS 9.4 (SAS Institute Inc., Cary, NC, USA) for Windows.

Due to the nature of the cognitive support tools used in the trial, blinding of participants as well as of research staff involved in the simulation sessions and video reviewers, is not possible. However, blinding of the statistician performing data analysis will be ensured. Data analysis is expected to be finalized by February 2022.

3.4 Outcomes

3.4.1 Primary outcome

The primary outcome of the study is the number of deviations from the PALS guidelines made by the teams during the management of a standardized simulated scenario of non-shockable PCA.

Deviations from PALS guideline recommendations are defined as delays and errors according to a novel checklist we derived from the previously published checklist by Wolfe et al[20], denominated c-DEV, by integrating it with evidence-based guidelines,[6-8,11] previously reported scoring tools,[40-43] and checklists.[37,44,45] We named our new modified checklist c-DEV15plus. It includes 15 items, which represent correct critical actions for paediatric resuscitation (Table 1).

Table 1: c-DEV15plus items for non-shockable PCA simulation scenario

1. CPR started within 30 seconds (s) from recognition of pulseless state
2. CPR board/rigid surface positioned underneath the manikin within 60 s from recognition of pulseless state
3. Compression/ventilation ratio 15:2
4. Help called (hospital emergency response system activated) within 60 s from recognition of pulseless state
5. Compressors switched more than once during CPR
6. ECG-monitoring started within 60 s from recognition of pulseless state
7. IV/IO access called within 60 s from recognition of pulseless state
8. First epinephrine called within 30 s from recognition of pulseless state
9. First epinephrine administered at the correct dose and dilution[a] and by the correct route (IV or IO), followed by a normal saline flush, while compressions are being performed, within 300 s (5 minutes) from recognition of pulseless state
10. Second epinephrine called between 3 and 5 minutes from the first administration of epinephrine
11. Second epinephrine administered at the correct dose and dilution[a] and by the correct route, followed by a normal saline flush, while compressions are being performed, within 5 min from the first epinephrine
12. Blood gas called during cardiac arrest
13. Reversible causes treated
14. Shock not administered
15. Medications other than adrenaline (e.g. amiodarone, lidocaine, atropine) not administered[b]

Abbreviations: CPR=cardiopulmonary resuscitation; s= seconds; ECG=electrocardiogram; IV= intravenous; IO=intraosseous; min= minutes. *Note*: [a] Correct dose of epinephrine is defined as 0.01 mg/kg (or a deviation from the correct weight dose of less than 10%); correct dilution of epinephrine is defined as 1:10.000 (0,1 mg/ml); [b] Administration of medications to treat identified reversible causes is not considered in this item.

Each item of the c-DEV15plus is scored either as 0, when the action is performed correctly and timely, as described in the item, or as 1, when the action is not undertaken, undertaken incorrectly, or with wrong timing and, in the event of drug administration, when the dose, duration or route of administration is wrong. The sum of the points attributed to the items represents the c-DEV15plus total score, hence ranging from 0 to 15, with higher scores corresponding to a higher number of deviations from the guidelines. Outcome assessors will score the scenarios through the c-DEV15plus tool using the data registered and coded by the video reviewers.

3.4.2 Secondary outcomes

The following secondary outcome measures will be collected:

- *Performance and time to accomplish critical resuscitation interventions* recommended by PALS guidelines.[6-11,20] The interventions evaluated will be: (i) cardiac arrest recognition (pulselessness); (ii) start of chest compressions; (iii) start of ventilation; (iv) use of a CPR board or a rigid surface underneath the manikin; (v) call for emergency team help; (vi) start of EKG monitor; (vi) first epinephrine administration; (vii) second epinephrine administration; and (viii) treatment of reversible causes of CA. Performance and time to accomplish critical interventions (in seconds) during resuscitation will be assessed and recorded by video reviewers in the study CRFs and will be analysed both as time from the beginning of the scenario and as time from the recognition of pulselessness.
- *Usability of the app*. To assess the PediAppRREST app usability, the team leaders of the intervention group will be administered one validated questionnaire, the System Usability Scale (SUS).[46,47] Further, the team leader will be asked to complete a questionnaire with open-ended questions.
- *Team leaders' workload* measured by the validated, multidimensional NASA-Task Load Index (NASA-TLX).[48,49] This tool includes six subscale scores that represent independent clusters of variables and different domains of the perceived workload: Mental, Physical, and Temporal Demands, Frustration, Effort, and Performance.
- *CPR quality* measured by the Skill Reporter (Laerdal ™), the internal software of the manikin. CPR quality is defined as: i) proportion of chest compressions with depth 50-60 mm; ii) chest compression fraction (the percentage of time during CA with chest compressions), iii) mean chest compression depth; and iv) mean chest compression rate, according to AHA standards.[7,50]

- Team *resuscitation performance* as evaluated with the Clinical Performance Tool (CPT).[40,41] The CPT is a validated scoring system designed based on PALS algorithms, through which sequence, timing, and quality of specific actions, during different simulated scenarios, can be assessed. The CPT section for the asystole will be used to evaluate teams' performance.

3.5 Intervention

3.5.1 Intervention arm - PediAppRREST app

The PediAppRREST, is an interactive, multimodal (audio-visual), "checklist" app, sequentially displaying prompts on recommended PCA management interventions.[37] It was specifically designed in 2019 to guide the team leader to perform resuscitation interventions in the sequence/timing and modality reported by the AHA PALS 2015 guidelines.[6-8]

The design and development of the app was guided by the results of a previous study conducted by our research team, which assessed deviations from guidelines in PCA simulation scenarios managed by paediatric residents.[38] The app was further refined following an iterative prototyping development approach with serial testing by our research staff, and according to the feedback provided by paediatric residents involved in a simulation-based pilot study.[37] The PediAppRREST app received a good usability evaluation and did not appear to increase team leaders' workload.[37]

Following the publication of the updated AHA PALS 2020 guidelines [11], the content and prompts of the app were checked against the guideline updated recommendations. Prompts to administer epinephrine as soon as possible and to guide post-arrest management were already provided by the app. The only content that required changing was the recommended ventilation rate for patients with an advanced airway from 1 breath every 6 seconds to 1 breath every 2 to 3 seconds. However, this last parameter is not included in the study outcomes, and it will not affect the results of our study.

3.5.2 Control arm - PALS pocket reference card

The AHA PALS pocket reference card is a 10 cm x 16.5 cm, full-colour, 2-sided, 6-panel card that shows the AHA treatment algorithms.[51] By providing a quick reference tool, it serves as a cognitive aid for healthcare providers who either direct or participate in the management of paediatric respiratory and/or cardiovascular emergencies, including CA. The PALS pocket reference card is used during the PALS course and in a variety of healthcare settings. Although there is no published evidence on its effectiveness, the PALS reference pocket card by summarizing the content and sequence of recommended interventions, is the cognitive aid most

widely used worldwide. Participants were provided the 2015 AHA PALS reference pocket card [51] until the new 2020 AHA PALS reference pocket card [52] was available and introduced in the study in 2021. Participants in this arm are also allowed to use a pocket calculator to compute medication dosages/dilution and a timepiece.

3.5.3 Control arm - no cognitive aid

Teams who are assigned to the CtrlPALS-group, are not able to use neither the PediAppRREST app nor the PALS pocket reference card to manage the simulated scenario. However, they will be allowed to use a pocket calculator to work out medication dosages/dilution and a timepiece, but no other cognitive tool.

3.6 Study procedures

Participant recruitment and assessment will take place over a 15-month period (September 2020 – December 2021). The University Hospital of Padua is the coordinating centre of the trial. Its research team will oversee all study procedures and processes during the simulation sessions to assure standardised high-quality procedures are carried out at each participating centre (Padua, Florence, Novara, Rome). At the trial simulation sessions, the research staff meet the residents, illustrate the study, answer any possible questions, re-assess eligibility criteria for each participant, and obtain informed consent for study participation and video recording.

During all trial sessions measures to prevent COVID-19 infection spread (physical distancing, hand hygiene, use of personal protective equipment during the scenario, temperature and health checks, contact tracing, surfaces/mannequin/equipment disinfection, etc.) are strictly followed for participants and research staff's safety.[53,54]

Before beginning the simulation session, all teams watch the same 20-minute briefing video about the study procedures and orientation to the setting, manikin, and equipment. This phase has also the aim of increasing participants' adherence to intervention protocols and study procedures.

After the briefing, each team progressively receives and opens its assigned sealed envelope, which contains the arm allocation (PediAppRREST or CtrlPALS+ or CtrlPALS-). Thereafter, all participants wear a sticker with the corresponding team identification number and personal identification code. Ten minutes before their assigned simulated scenario each team is informed that the scenario will be about a PCA case, without specifying any further detail. The teams assigned to the intervention arm (PediAppRREST) watch a 5-minute tutorial video about the app and its use, prepared ad-hoc for this study. They are also given five minutes to practically

familiarize with the tablet and the app. The teams in the CtrlPALS+ arm are given five minutes before the scenario to familiarize with the PALS pocket algorithm card, while teams in the CtrlPALS- arm are given five minutes to discuss how to manage the scenario without any PALS-related cognitive aid.

Subsequently, each team participates in a 10-minute standardized intermediate-fidelity simulated scenario of non-shockable PCA caused by hypovolemia and hypoglycemia. A non-shockable rhythm was chosen for the simulated scenario because it is the most common initial cardiac arrest rhythm detected in PCA.[55] The setting, the equipment set up, the scenario, and the manikin (Resusci Junior QCPR Laerdal ™ whose head is replaced with the MegaCodeKid Laerdal ™ manikin's head, on which advanced air management can be performed) are the same for all the centres. The scenario is introduced by a standardized video where an actress, playing the patient's (manikin's) mother, provides essential clinical information (further scenario details are included in the Appendix 1). The scenarios are conducted off-site, in rooms set up to resemble the Emergency Department Shock room including regularly available equipment. The simulation rooms are set up in a standardized fashion between participating sites. Every team works with one confederate nurse and can call confederate consultants on the phone, who answer based on a standardized script. During the scenario, the team can speak with a facilitator, who is a member of the research team and answers participants' questions following a pre-determined script.

All the scenarios are video-recorded by two different fixed cameras with standardized positions that point to the team and to the monitor. An additional camera captures the actions of the team leaders who use the PediAppRREST app, framing the screen of the tablet. Only the videos from the two fixed cameras will be evaluated by the reviewers. The videos of the team leaders' actions with the tablet will be examined only by the research team to assess potential bugs in the app or challenges with its use.

After the scenario, all participants complete a demographic survey reporting their sex, age, year and type of residency program, experience in simulation and resuscitation, and time from PALS/P-BLS/BLS/ALS/ACLS certification. In addition, all the team leaders complete the NASA-TLX and the team leaders in the intervention arm also evaluate the app usability, by completing the SUS. Subsequently, all participants participate in a 15-minute debriefing during which they receive feedback about their performance, and the teams in the intervention arm are able to provide feedback about the app. During this phase, the debriefer reports the feedback received on the app in a specific CRF.

The videos of the scenarios will be evaluated by two independent, and previously trained, reviewers expert in paediatric emergency medicine and simulation. Training of the reviewers, on

RCT-unrelated PCA simulation videos, will be conducted by the principal investigator (FC), until the reviewers will reach at least 80% inter-rater agreement with the principal investigator. No more than two weeks will lapse between the training and the assessment of the RCT videos. The reviewers will use a standardized data collection form where actions performed by the team and time to performance will be assessed and recorded. Disagreements between reviewers will be resolved by consensus with a third independent reviewer, expert in paediatric emergency medicine and simulation. Inter-rater reliability between reviewers will be monitored and reported. Data pertaining the CPR quality from the manikin software will be extracted and recorded on a specific CRF. The scores on the c-DEV15plus, and all the secondary outcomes will be calculated by outcome assessors based on the data coded and reported by the video reviewers on the CRFs.

3.7 Data Collection and management

Participants' data are pseudo-anonymized by assigning a unique code to each participant. Data pertaining the participants' information, video reviews, and the outcomes investigated in the trial, is recorded using pseudo-anonymized CRFs. Completed CRFs are checked for completeness and accuracy by the principal investigators.

All CRFs data is securely stored in electronic databases created using REDCap (Research Electronic Data Capture), a browser-based, metadata-driven software solution and workflow methodology used to design clinical and translational research secure password protected databases (REDCap, Vanderbilt University, Nashville, TN, USA).[56] Only the principal investigators and the study statistician will have access to the final trial dataset.

Data will be analysed by the study statistician (ACF) who will be blinded to group allocation coding.

3.8 Statistical Analysis

3.8.1 Sample size calculation

We calculated sample size on the basis of the results obtained during the previous observational simulation-based study and the pilot study that tested the app usability.[37,38] Based on the preliminary results from these studies, using a single factor ANOVA model, 29 scenarios per each of the three groups (PediAppRREST, CtrlPALS+, CtrlPALS-) are necessary to detect a difference of at least 3.00 points on the c-DEV15plus scale using the Tukey-Kramer

(Pairwise) multiple comparison procedure at a 5% significance level and 80% power. The common standard deviation within a group is assumed to be 2.20.

In consideration that some possible technical problems with video-recording or other study procedures could occur, we aim to increase the recruitment of participating teams by 20% per arm, to compensate for loss of statistical power due to a potential insufficient sample size. Hence, we plan to have 35 scenarios per arm, for a total of 105 scenarios, which will include overall 315 residents divided in teams of three.

3.8.2 Data analysis plan

The results will be summarized for each study group with counts and percentages for categorical variables, mean and standard deviation (SD) or median and interquartile range for quantitative variables, as appropriate. The normality of the quantitative variables will be checked with the Shaphiro-Wilk test.

The c-DEV15plus scale, the performance and time to accomplish specific resuscitation interventions, NASA-TLX, CPT and cardiopulmonary resuscitation metrics will be compared between groups with one-way ANOVA model, followed by the Tukey-Kramer multiple comparisons adjustment procedure in case of statistical significance.

The outcomes will also be analysed with a linear mixed model considering the team as a cluster to evaluate the influence of participants' characteristics on the outcome. To take into account the correlation of the observations within a team, we will specify an undetermined correlation matrix. In case of a not normal distribution of the model residuals, we will proceed with a transformation in order to normalize the distribution.

Both intention-to-treat and per-protocol analyses will be performed.

3.9 Patient and Public Involvement

No patient involved.

4. ETHICS AND DISSEMINATION

The design of this study complies with the Declaration of Helsinki ethical principles, Good Clinical Practice standards, and European Union general data protection regulation on scientific research. Participation into the study is on a voluntary basis and bears no academic or professional consequences on the medical residents. A written informed consent to take part into the study is obtained from each participant. The Human Ethics Committee (HEC) of the University Hospital of Padova, coordinating centre of the trial, deemed the trial to be a negligible risk study and approved it through an expedited review process.

The results of the study will be disseminated in peer-reviewed journals, national and international scientific conferences, and medical residency training programs educational sessions. After publication of the study results, the PediAppRREST app will be released exclusively to institutions/health care professionals, upon request.

Any adverse event will be communicated to the principal investigators and recorded in the participant CRF. In the extremely unlikely event of a serious adverse event, the principal investigators will be informed immediately and the HEC will be notified within 24-72 hours of occurrence.

This trial is registered in clinicaltrials.gov, ID: NCT04619498 (November 6, 2020).

5. AUTHORS' CONTRIBUTIONS

Drs Silvia Bressan, Francesco Corazza, Deborah Snijders, Marta Arpone, Valentina Stritoni, Pier Luigi Ingrassia, Marco De Luca, Luca Tortorolo, Anna Chiara Frigo, and Liviana Da Dalt conceptualized the study. Drs Silvia Bressan and Francesco Corazza designed the study and its procedures, with input from Dr Adam Cheng. Drs Francesco Corazza and Marta Arpone wrote the first draft of the protocol and contributed equally to this paper. All authors contributed to the revision of the protocol and editing of the manuscript.

6. FUNDING STATEMENT

This research has received funding support from internal grants from the University of Padova (BIRD 191291).

7. COMPETING INTERESTS STATEMENT

The authors declare that they have no competing interests.

8. REFERENCES

1. Donoghue AJ, Nadkarni V, Berg RA, et al. Out-of-hospital pediatric cardiac arrest: an epidemiologic review and assessment of current knowledge. *Ann Emerg Med* 2005;46(6):512-22. doi: 10.1016/j.annemergmed.2005.05.028
2. Knudson JD, Neish SR, Cabrera AG, et al. Prevalence and outcomes of pediatric in-hospital cardiopulmonary resuscitation in the United States: an analysis of the Kids' Inpatient Database. *Crit Care Med* 2012;40(11):2940-44. doi: 10.1097/CCM.0b013e31825feb3f
3. Nehme Z, Namachivayam S, Forrest A, et al. Trends in the incidence and outcome of paediatric out-of-hospital cardiac arrest: A 17-year observational study. *Resuscitation* 2018;128:43-50. doi: 10.1016/j.resuscitation.2018.04.030
4. Reis AG, Nadkarni V, Perondi MB, et al. A prospective investigation into the epidemiology of in-hospital pediatric cardiopulmonary resuscitation using the international Utstein reporting style. *Pediatrics* 2002;109(2):200-09. doi: 10.1542/peds.109.2.200
5. Tibballs J, Kinney S. A prospective study of outcome of in-patient paediatric cardiopulmonary arrest. *Resuscitation* 2006;71(3):310-18. doi: 10.1016/j.resuscitation.2006.05.009
6. Atkins DL, Berger S, Duff JP, et al. Part 11: pediatric basic life support and cardiopulmonary resuscitation quality: 2015 American Heart Association guidelines update for cardiopulmonary resuscitation and emergency cardiovascular care. *Circulation* 2015;132(18_suppl_2):S519-25. doi: 10.1161/CIR.0000000000000265
7. De Caen AR, Berg MD, Chameides L, et al. Part 12: pediatric advanced life support: 2015 American Heart Association guidelines update for cardiopulmonary resuscitation and emergency cardiovascular care. *Circulation* 2015;132(18_suppl_2):S526-42. doi: 10.1161/CIR.0000000000000266
8. Duff JP, Topjian AA, Berg MD, et al. 2019 American Heart Association focused update on pediatric advanced life support: an update to the American Heart Association guidelines for cardiopulmonary resuscitation and emergency cardiovascular care. *Pediatrics* 2020;145(1):e20191361. doi: 10.1542/peds.2019-1361
9. Maconochie IK, Bingham R, Eich C, et al. European Resuscitation Council guidelines for resuscitation 2015: Section 6. Paediatric life support. *Resuscitation* 2015;95:223-48. doi: 10.1016/j.resuscitation.2015.07.028
10. Maconochie IK, de Caen AR, Aickin R, et al. Part 6: Pediatric basic life support and pediatric advanced life support: 2015 International Consensus on Cardiopulmonary Resuscitation and Emergency Cardiovascular Care Science with Treatment

Recommendations. *Resuscitation* 2015;95:e147-68. doi: 10.1016/j.resuscitation.2015.07.044

11. Topjian AA, Raymond TT, Atkins D, et al. Part 4: Pediatric basic and advanced life support: 2020 American Heart Association guidelines for cardiopulmonary resuscitation and emergency cardiovascular care. *Circulation* 2020;142(16_suppl_2):S469-S523. doi: 10.1161/CIR.0000000000000901
12. Cheng A, Magid DJ, Auerbach M, et al. Part 6: Resuscitation education science: 2020 American Heart Association guidelines for cardiopulmonary resuscitation and emergency cardiovascular care. *Circulation* 2020;142(16_suppl_2):S551-79. doi: 10.1161/CIR.0000000000000903
13. Abella BS, Alvarado JP, Myklebust H, et al. Quality of cardiopulmonary resuscitation during in-hospital cardiac arrest. *JAMA* 2005;293(3):305-10. doi: 10.1001/jama.293.3.305
14. Sutton RM, Niles D, French B, et al. First quantitative analysis of cardiopulmonary resuscitation quality during in-hospital cardiac arrests of young children. *Resuscitation* 2014;85(1):70-74. doi: 10.1016/j.resuscitation.2013.08.014
15. Sutton RM, Case E, Brown SP, et al. A quantitative analysis of out-of-hospital pediatric and adolescent resuscitation quality--A report from the ROC epistry-cardiac arrest. *Resuscitation* 2015;93:150-57. doi: 10.1016/j.resuscitation.2015.04.010
16. Niles DE, Duval-Arnould J, Skellett S, et al. Characterization of pediatric in-hospital cardiopulmonary resuscitation quality metrics across an international resuscitation collaborative. *Pediatr Crit Care Med* 2018;19(5):421-32. doi: 10.1097/pcc.0000000000001520
17. Cheng A, Hunt EA, Grant D, et al. Variability in quality of chest compressions provided during simulated cardiac arrest across nine pediatric institutions. *Resuscitation* 2015;97:13-19. doi: 10.1016/j.resuscitation.2015.08.024
18. Hunt EA, Vera K, Diener-West M, et al. Delays and errors in cardiopulmonary resuscitation and defibrillation by pediatric residents during simulated cardiopulmonary arrests. *Resuscitation* 2009;80(7):819-25. doi: 10.1016/j.resuscitation.2009.03.020
19. Labrosse M, Levy A, Donoghue A, et al. Delays and errors among pediatric residents during simulated resuscitation scenarios using Pediatric Advanced Life Support (PALS) algorithms. *Am J Emerg Med* 2015;33(10):1516-18. doi: 10.1016/j.ajem.2015.07.049

20. Wolfe HA, Morgan RW, Zhang B, et al. Deviations from AHA guidelines during pediatric cardiopulmonary resuscitation are associated with decreased event survival. *Resuscitation* 2020;149:89-99. doi: 10.1016/j.resuscitation.2020.01.035
21. McEvoy MD, Field LC, Moore HE, et al. The effect of adherence to ACLS protocols on survival of event in the setting of in-hospital cardiac arrest. *Resuscitation* 2014;85(1):82-87. doi: 10.1016/j.resuscitation.2013.09.019
22. Hunt EA, Heine M, Shilkofski NS, et al. Exploration of the impact of a voice activated decision support system (VADSS) with video on resuscitation performance by lay rescuers during simulated cardiopulmonary arrest. *Emerg Med J* 2015;32(3):189-94. doi: 10.1136/emermed-2013-202867
23. Metelmann B, Metelmann C, Schuffert L, et al. Medical correctness and user friendliness of available apps for cardiopulmonary resuscitation: systematic search combined with guideline adherence and usability evaluation. *JMIR Mhealth Uhealth* 2018;6(11):e190. doi: 10.2196/mhealth.9651
24. Low D, Clark N, Soar J, et al. A randomised control trial to determine if use of the iResus(c) application on a smart phone improves the performance of an advanced life support provider in a simulated medical emergency. *Anaesthesia* 2011;66(4):255-62. doi: 10.1111/j.1365-2044.2011.06649.x
25. Lelaidier R, Balanca B, Boet S, et al. Use of a hand-held digital cognitive aid in simulated crises: the MAX randomized controlled trial. *Br J Anaesth* 2017;119(5):1015-21. doi: 10.1093/bja/aex256
26. Donzé P, Balanca B, Lilot M, et al. 'Read-and-do' response to a digital cognitive aid in simulated cardiac arrest: the Medical Assistance eXpert 2 randomised controlled trial. *Br J Anaesth* 2019;123(2):e160-63. doi: 10.1016/j.bja.2019.04.049
27. Field LC, McEvoy MD, Smalley JC, et al. Use of an electronic decision support tool improves management of simulated in-hospital cardiac arrest. *Resuscitation* 2014;85(1):138-42. doi: 10.1016/j.resuscitation.2013.09.013
28. Hawkes GA, Murphy G, Dempsey EM, et al. Randomised controlled trial of a mobile phone infant resuscitation guide. *J Paediatr Child Health* 2015;51(11):1084-88. doi: 10.1111/jpc.12968
29. Siebert JN, Ehrler F, Gervaix A, et al. Adherence to AHA guidelines when adapted for augmented reality glasses for assisted pediatric cardiopulmonary resuscitation: a randomized controlled trial. *J Med Internet Res* 2017;19(5):e183. doi: 10.2196/jmir.7379

30. Siebert JN, Ehrler F, Combescure C, et al. A mobile device application to reduce medication errors and time to drug delivery during simulated paediatric cardiopulmonary resuscitation: a multicentre, randomised, controlled, crossover trial. *Lancet Child Adolesc Health* 2019;3(5):303-11. doi: 10.1016/s2352-4642(19)30003-3
31. Siebert JN, Lacroix L, Cantais A, et al. The impact of a tablet app on adherence to American Heart Association guidelines during simulated pediatric cardiopulmonary resuscitation: randomized controlled trial. *J Med Internet Res* 2020; 22(5):e17792. doi: 10.2196/17792
32. Lin YY, Chiang WC, Hsieh MJ, et al. Quality of audio-assisted versus video-assisted dispatcher-instructed bystander cardiopulmonary resuscitation: A systematic review and meta-analysis. *Resuscitation* 2018; 123:77-85. doi: 10.1016/j.resuscitation.2017.12.010
33. Kirkbright S, Finn J, Tohira H, et al. Audiovisual feedback device use by health care professionals during CPR: a systematic review and meta-analysis of randomised and non-randomised trials. *Resuscitation* 2014;85(4):460-471. doi: 10.1016/j.resuscitation.2013.12.012
34. Lakomek F, Lukas RP, Brinkrolf P, et al. Real-time feedback improves chest compression quality in out-of-hospital cardiac arrest: A prospective cohort study. *PLoS One* 2020;15(2):e0229431. doi: 10.1371/journal.pone.0229431
35. Lin Y, Cheng A, Grant VJ, et al. Improving CPR quality with distributed practice and real-time feedback in pediatric healthcare providers - A randomized controlled trial. *Resuscitation* 2018;130:6-12. doi: 10.1016/j.resuscitation.2018.06.025
36. Wagner M, Bibl K, Hrdliczka E, et al. Effects of feedback on chest compression quality: a randomized simulation Study. *Pediatrics* 2019;143(2):20182441. doi: 10.1542/peds.2018-2441
37. Corazza F, Snijders D, Arpone M, et al. Development and usability of a novel interactive tablet app (PediAppRREST) to support the management of pediatric cardiac arrest: pilot high-fidelity simulation-based study. *JMIR MHealth UHealth* 2020;8(10):e19070. doi: 10.2196/19070
38. Stritoni V, Martinolli F, Daverio M, et al. Gaps in the management of nonshockable paediatric cardiac arrest by paediatric residents in simulated scenario. Proceedings of the Third European Pediatric Resuscitation and Emergency Medicine Conference (PREM). Ghent, Belgium, 2019 May 23-24.

39. Cheng A, Kessler D, Mackinnon R, et al. Reporting guidelines for health care simulation research: extensions to the CONSORT and STROBE statements. *Simul Healthc* 2016;11(4):238-48. doi: 10.1097/SIH.0000000000000150
40. Levy A, Donoghue A, Bailey B, et al. External validation of scoring instruments for evaluating pediatric resuscitation. *Simul Healthc* 2014;9(6):360-69. doi: 10.1097/SIH.0000000000000052
41. Donoghue A, Ventre K, Boulet J, et al. Design, implementation, and psychometric analysis of a scoring instrument for simulated pediatric resuscitation: a report from the EXPRESS pediatric investigators. *Simul Healthc* 2011;6(2):71-77. doi: 10.1097/SIH.0b013e31820c44da
42. Brett-Fleegler MB, Vinci RJ, Weiner DL, et al. A simulator-based tool that assesses pediatric resident resuscitation competency. *Pediatrics* 2008;121(3):e597-603. doi: 10.1542/peds.2005-1259
43. Reid J, Stone K, Brown J, et al. The Simulation Team Assessment Tool (STAT): development, reliability and validation. *Resuscitation* 2012;83(7):879-86. doi: 10.1016/j.resuscitation.2011.12.012
44. Arriaga AF, Bader AM, Wong JM, et al. Simulation-based trial of surgical-crisis checklists. *N Engl J Med* 2013;368(3):246-53. doi: 10.1056/NEJMsa1204720
45. McEvoy MD, Smalley JC, Nietert PJ, et al. Validation of a detailed scoring checklist for use during advanced cardiac life support certification. *Simul Healthc* 2012;7(4):222-35. doi: 10.1097/SIH.0b013e3182590b07
46. Lewis JR. The System Usability Scale: Past, Present, and Future. *Hum-Comput Interact* 2018;34(7):577-90. doi: 10.1080/10447318.2018.1455307
47. Brooke J. SUS: A ‘quick and dirty’ usability scale. In: Jordan PW, Thomas B, Weerdmeester A, et al., eds. Usability evaluation in industry. London Taylor & Francis 1996: 189-94.
48. Hart SG. Nasa-Task Load Index (NASA-TLX); 20 Years Later. *Proc. Hum. Factors Ergon. Soc. Annu. Meet* 2006;50(9):904-08. doi: 10.1177/154193120605000909
49. Cao A, Chintamani KK, Pandya AK, et al. NASA TLX: software for assessing subjective mental workload. *Behav Res Methods* 2009;41(1):113-17. doi: 10.3758/BRM.41.1.113
50. Cheng A, Duff JP, Kessler D, et al. Optimizing CPR performance with CPR coaching for pediatric cardiac arrest: A randomized simulation-based clinical trial. *Resuscitation* 2018;132:33-40. doi: 10.1016/j.resuscitation.2018.08.021

51. American Heart Association and American Academy of Pediatrics. Pediatric advanced life support. Provider manual and pocket card. 2016. United States of America.
52. American Heart Association. Pediatric advanced life support. Provider manual and pocket card. 2020. United States of America.
53. Ingrassia PL, Capogna G, Diaz-Navarro C, et al. COVID-19 crisis, safe reopening of simulation centres and the new normal: food for thought. *Adv Simul* (Lond) 2020;5:13. doi: 10.1186/s41077-020-00131-3
54. Park CS, Clark L, Gephardt G, et al. Manifesto for healthcare simulation practice. *BMJ Simul Technol Enhanc Learn* 2020;6:365-68. doi: 10.1136/bmjstel-2020-000712
55. Girotra S, et al. Survival trends in pediatric in-hospital cardiac arrests: an analysis from Get With the Guidelines-Resuscitation. *Circ Cardiovasc Qual Outcomes*. 2013;6(1):42-49. doi:10.1161/CIRCOUTCOMES.112.967968
56. Harris PA, Taylor R, Minor BL, et al. The REDCap consortium: Building an international community of software platform partners. *J Biomed Inform* 2019;95:103208. doi: 10.1016/j.jbi.2019.103208

9. APPENDIX 1

Simulation scenario

Case scenario: a case of non-shockable paediatric cardiac arrest caused by hypovolemia and hypoglycaemia.
Team: three paediatric residents and one confederate nurse.
Setting: off-site, in a room set reproducing the environment and the equipment of a paediatric emergency department shock room.

Introduction: A 4-year-old child (22 kg weight) is brought to the Emergency Department by his mother; she refers diarrhoea and vomiting in the past 5 days. Today the boy has been lethargic and difficult to wake up; the triage nurse brings him straight to the shock room.

Information about the clinical case is provided to participants through a video where an actress plays the role of a mother who brings her child to the Paediatric Emergency Department.

The following information about the child are conveyed through the video:

- Age: 4 years;
- Weight: 22 Kg;
- Signs/Symptoms: vomiting and diarrhoea for the previous 5 days, minimal fluid intake for the past 2-3 days;
- Allergies: no allergies;
- Medications: no medications;
- Past history: previously healthy child;
- Last meal: more than 48 hours before;
- Events: child has been sleeping for the previous 2 hours, not responding to physical stimulation.

The scenario starts with a pulseless unconscious child who is suffering a cardiac arrest characterised by pulseless electrical activity (PEA) rhythm for 2 minutes, followed by asystole.

Return of spontaneous circulation (ROSC) is achieved if at least two correct doses of epinephrine are administered timely and hypovolemia +/- hypoglycaemia are addressed. The scenario runs for 10 minutes following the end of the introductive video, regardless of the actions performed by the team.
