# Supplementary material for: Effectiveness of a Novel Tablet Application in Reducing Guideline Deviations During Pediatric Cardiac Arrest: A Randomized Clinical Trial
Source: JAMA Netw Open. 2023 Aug 3;6(8):e2327272. doi: 10.1001/jamanetworkopen.2023.27272 (PMC10401301; doi:10.1001/jamanetworkopen.2023.27272)
Supplement: Supplement 2. — eAppendix 1. Simulation Scenario eAppendix 2. Further Details Regarding Statistical Analysis eTable 1. Teams’ Distributions Between Study Site and Residency Program eTable 2. Subanalysis of Error Score by Residency Program eTable 3. Performance of Single Critical Resuscitation Actions eTable 4. Time to Perform Single Critical Resuscitation Actions eTable 5. Single Items of Clinical Performance Tool eTable 6. Issues in Using the PediAppRREST App and Proposed Solutions eTable 7. Single Items of Raw NASA Task Load Index [file jamanetwopen-e2327272-s002.pdf]

Supplemental Online Content

Corazza F, Arpone M, Tardini G, et al. Effectiveness of a novel tablet application in reducing guideline deviations during pediatric cardiac arrest: a randomized clinical trial. *JAMA Netw Open*. 2023;6(8):e2327272. doi:10.1001/jamanetworkopen.2023.27272

eAppendix 1. Simulation Scenario

eAppendix 2. Further Details Regarding Statistical Analysis

eTable 1. Teams' Distributions Between Study Site and Residency Program

eTable 2. Subanalysis of Error Score by Residency Program

eTable 3. Performance of Single Critical Resuscitation Actions

eTable 4. Time to Perform Single Critical Resuscitation Actions

eTable 5. Single Items of Clinical Performance Tool

eTable 6. Issues in Using the PediAppRREST App and Proposed Solutions

eTable 7. Single Items of Raw NASA Task Load Index

This supplemental material has been provided by the authors to give readers additional information about their work.

© 2023 Corazza F et al. *JAMA Network Open.*

eAppendix 1. Simulation Scenario

Replica of case scenario presented as supplementary material with the protocol paper Corazza F et al, BMJ Open 2021

Case scenario: a case of non-shockable pediatric cardiac arrest caused by hypovolemia and hypoglycemia.
Team: three pediatric residents and one confederate nurse.
Setting: off-site, in a room set reproducing the environment and the equipment of a pediatric emergency department shock room.

Introduction: A 4-year-old child (22 kg weight) is brought to the Emergency Department by his mother; she refers diarrhea and vomiting in the past 5 days. Today the boy has been lethargic and difficult to wake up; the triage nurse brings him straight to the shock room.

Information about the clinical case is provided to participants through a video where an actress plays the role of a mother who brings her child to the Pediatric Emergency Department.

The following information about the child are conveyed through the video:

- Age: 4 years;
- Weight: 22 Kg;
- Signs/Symptoms: vomiting and diarrhea for the previous 5 days, minimal fluid intake for the past 2-3 days;
- Allergies: no allergies;
- Medications: no medications;
- Past history: previously healthy child;
- Last meal: more than 48 hours before;
- Events: child has been sleeping for the previous 2 hours, not responding to physical stimulation.

The scenario starts with a pulseless unconscious child who is suffering a cardiac arrest characterized by pulseless electrical activity (PEA) rhythm for 2 minutes, followed by asystole.

Return of spontaneous circulation (ROSC) is achieved if cardiopulmonary resuscitation was correctly performed, at least two correct doses of epinephrine are administered timely and hypovolemia +/- hypoglycemia are addressed. The scenario runs for 10 minutes following the end of the introductive video, regardless of the actions performed by the team.

© 2023 Corazza F et al. *JAMA Network Open.*

eAppendix 2. Further Details Regarding Statistical Analysis

Data regarding Error total score, Clinical Performance Tool (CPT) total score, Raw NASA-Task Load Index (R-TLX) total score and the single items of the R-TLX score, chest compression quality, and time to perform single resuscitation actions were analyzed with generalized linear models considering the ANOVA for quantitative variables; participants' residency program were analyzed with generalized logit for multinomial non-ordinal data; individual items of the CPT score, year of residency, and the number of simulation during the last year were analyzed with cumulative logit for multinomial ordinal data; and individual items of Error score, the item 7 of the CPT, performance of individual resuscitation actions, and the rest of the participants' characteristics were analyzed with binomial distribution for binary data.

© 2023 Corazza F et al. *JAMA Network Open.*

eTable 1. Teams' Distributions Between Study Site and Residency Program

**Teams included, n**	**Pediatrics**	**Anesthesiology**	**Emergency Medicine**	**Total**
**Padua**	30	9	9	48
**Florence**	19	0	0	19
**Rome**	10	3	4	17
**Novara**	11	4	1	16
**Total**	70	16	14	100

Abbreviations: n: number of teams

© 2023 Corazza F et al. *JAMA Network Open.*

eTable 2. Subanalysis of Error Score by Residency Program

**Residency Program**		**PediAppRREST**	**PALS**	**Null control**	**P value**	**Tukey-Kramer adjusted pairwise comparisons P value**		
						**PediAppRREST vs PALS**	**PediAppRREST vs null control**	**PALS vs null control**
Pediatrics	n	22	25	23				
	Error score, M (SD)	3.5 (2.1)	6.6 (1.8)	6.2 (1.7)	**<0.001**	**<0.001**	**<0.001**	0.72
Anesthesiology	n	6	6	4				
	Error score, M (SD)	3.2 (1.5)	6.2 (2.2)	4.8 (1.0)	**0.03**	**0.03**	0.36	0.43
Emergency medicine	n	4	4	6				
	Error score, M (SD)	3.5 (2.1)	5.5 (1.3)	6.0 (1.3)	0.08	na	na	na

Abbreviations: M = mean; n= number of teams, na = not applicable; PALS= Pediatric Advanced Life Support, SD= Standard Deviation.

© 2023 Corazza F et al. *JAMA Network Open.*

eTable 3. Performance of Single Critical Resuscitation Actions

	**PediAppRREST (n=32)**	**PALS (n=35)**	**Null control (n=33)**	**P value**
Pulse assessment, n (%)	29 (90.6)	32 (91.4)	30 (90.9)	0.99
Central pulse assessment, n (%)	28 (96.6)	26 (81.3)	22 (73.3)	**0.01**[a]
Recognition of cardiac arrest, n (%)	32 (100.0)	35 (100.0)	33 (100.0)	-
Call for help, n (%)	26 (81.3)	16 (45.7)	24 (72.7)	**0.005**[b]
EKG monitoring, n (%)	32 (100.0)	35 (100.0)	33 (100.0)	-
Start CPR, n (%)	32 (100.0)	35 (100.0)	33 (100.0)	-
Correct compression:ventilation ratio, n (%)	31 (96.9)	33 (94.3)	28 (84.8)	0.23
CPR board placement/rigid surface positioning, n (%)	25 (78.1)	7 (20.0)	9 (27.3)	**<0.001**[c]
Start ventilation, n (%)	32 (100.0)	35 (100.0)	33 (100.0)	-
IV/IO access placement, n (%)	32 (100.0)	35 (100.0)	33 (100.0)	-
Administration of first epinephrine, n (%)	32 (100.0)	35 (100.0)	33 (100.0)	-
Correct dose of epinephrine, n (%)*	30 (93.8)	30 (85.7)	27 (81.8)	0.27
Correct dilution of epinephrine, n (%)*	31 (96.9)	32 (91.4)	31 (93.9)	0.61
Saline flush after administration of first epinephrine, n (%)*	28 (87.5)	12 (34.3)	9 (27.3)	**<0.001**[d]
Administration of second epinephrine, n (%)	29 (90.6)	35 (100.0)	31 (93.9)	0.62
Correct dose of second epinephrine, n (%)*	29 (100.0)	30 (85.7)	24 (77.4)	0.39
Correct dilution for second epinephrine, n (%)*	29 (100.0)	32 (91.4)	29 (93.5)	0.74
Saline flush after administration of second epinephrine, n (%)*	26 (89.7)	9 (25.7)	10 (32.3)	**<0.001**[e]
Rotation of compressors, n (%)	32 (100.0)	26 (74.3)	28 (84.8)	0.28
Number of rotations of compressors, mean (SD)	2.2 (0.9)	1.3 (1.2)	2.1 (1.3)	**0.004**[f]
Call for blood gas, n (%)	30 (93.8)	27 (77.1)	29 (87.9)	0.14
Call for blood glucose, n (%)	25 (78.1)	16 (45.7)	18 (54.5)	**0.01**[g]
Consider advanced airway management, n (%)	18 (56.3)	24 (68.6)	19 (57.6)	0.51
Ask for history, n (%)	24 (75.0)	16 (45.7)	20 (60.6)	**0.04**[h]
Treatment of one reversible cause, n (%)	30 (93.8)	31 (88.6)	33 (100.0)	0.37
Treatment of hypovolemia, n (%)	24 (75.0)	30 (85.7)	30 (90.9)	0.23
Correct treatment of hypovolemia, n (%)	24 (100.0)	26 (89.7)	27 (93.1)	0.64
Treatment of hypoglycemia, n (%)	22 (68.8)	12 (34.3)	17 (51.5)	**0.01**[i]
Correct treatment of hypoglycemia, n (%)	21 (95.5)	10 (83.3)	10 (62.5)	**0.04**[j]
Administration of other medicaments, n (%)	1 (3.1)	2 (5.7)	1 (3.0)	0.84
Defibrillation performed, n (%)	1 (3.1)	1 (2.9)	0 (0.0)	0.46
ROSC achieved, n (%)	25 (78.1)	10 (28.6)	6 (18.2)	**<0.001**[k]

Abbreviations: CPR= cardiopulmonary resuscitation, EKG= electrocardiogram, IO= intraosseous, IV= intravenous, n= number of teams, PALS= Pediatric Advanced Life Support, ROSC= Return of Spontaneous Circulation, SD= Standard Deviation, %= percentage.

© 2023 Corazza F et al. *JAMA Network Open.*

Note: *Correct dose of epinephrine is defined as 0.01 mg/kg (or a deviation from the correct weight dose of less than 10%); correct dilution of epinephrine is defined as 0.1 mg/mL (1:10.000); correct modality of administration is defined as correct dose, dilution and by the correct route (IV/IO), followed by a normal saline flush, while chest compressions are being performed.

Pairwise comparisons (p value): [a]PediaAppRREST vs PALS p=0.12, PediAppRREST vs null control **p=0.03**, PALS vs null control p=0.74;
[b] PALS vs null control **p=0.04**, PediAppRREST vs CtrlPALS- p=0.69, PALS vs null control p=0.05;
[c]PediaAppRREST vs PALS **p<0.001**, PediAppRREST vs null control **p<0.001**, PALS vs null control p=0.76;
[d]PediaAppRREST vs PALS **p<0.001**, PediAppRREST vs null control **p<0.001**, PALS vs null control p=0.81;
[e]PediAppRREST vs PALS **p<0.001**, PediAppRREST vs null control **p<0.001**, PALS vs null control p=0.83;
[f]PediAppRREST vs PALS **p=0.007**, PediAppRREST vs null control p=0.94, PALS vs null control **p=0.02**;
[g]PediAppRREST vs PALS **p=0.01**, PediAppRREST vs null control p=0.10, PALS vs null control p=0.75;
[h]PediAppRREST vs PALS **p=0.03**, PediAppRREST vs null control p=0.42, PALS vs null control p=0.43;
[i]PediAppRREST vs PALS **p=0.009**, PediAppRREST vs null control p=0.32, PALS vs null control p=0.32;
[j]PediAppRREST vs PALS p=0.56, PediAppRREST vs null control **p=0.04**, PALS vs null control p=0.41;
[k]PediAppRREST vs PALS **p<0.001**, PediAppRREST vs null control **p<0.001**, PALS vs null control p=0.57.

© 2023 Corazza F et al. *JAMA Network Open.*

eTable 4. Time to Perform Single Critical Resuscitation Actions

	**PediAppRREST (n=32)**	**PALS (n=35)**	**Null control (n=33)**	**P value**
Time to pulse assessment (s), M (SD);<br>Median (IQR)	32.7 (16.1)<br>30.0 (19.0-41.0)	45.3 (27.3)<br>33.0 (25.5 – 63.5)	31.2 (20.2)<br>25.5 (18.0 – 38.0)	**0.02**[a]
Time to recognition of CA (s), M (SD);<br>Median (IQR)	44.8 (20.9)<br>42.0 (24.5 – 56.5)	53.0 (25.0)<br>50.0 (32.0 – 73.0)	43.9 (28.1)<br>36.0 (26.0 – 47.0)	0.26
Time to call for help (s), M (SD);<br>Median (IQR)	110.6 (94.1)<br>88.5 (56.0 – 107.0)	284.0 (140.5)<br>275.5 (192.5 – 350.5)	298.3 (137.0)<br>272.5 (204.0 – 388.5)	**<0.001**[b]
Time to call for help from recognition of CA (s), M (SD);<br>Median (IQR)	66.1 (91.0)<br>39.5 (24.0 – 65.0)	232.8 (138.1)<br>228.0 (130.0 – 300.5)	249.6 (137.6)<br>210.5 (166.5 – 342.0)	**<0.001**[c]
Time to start EKG monitoring (s), M (SD);<br>Median (IQR)	61.5 (32.0)<br>49.0 (39.0 – 85.0)	67.0 (47.2)<br>55.0 (32.0 – 90.0)	46.3 (22.0)<br>42.0 (30.0 – 59.0)	0.05
Time to start EKG monitoring from recognition of CA (s), M (SD);<br>Median (IQR)	17.2 (36.9)<br>18.5 (-6.5 – 41.5)	14.3 (51.4)<br>3.0 (-22.0 – 45.0)	2.6 (37.6)<br>0.0 (-12.0 – 26.0)	0.35
Time to start CPR (s), M (SD);<br>Median (IQR)	62.3 (29.0)<br>55.0 (39.5 – 75.5)	65.3 (28.3)<br>64.0 (39.0 – 83.0)	52.2 (28.3)<br>44.0 (34.0 – 58.0)	0.15
Time to CPR board placement/rigid surface positioning (s), M (SD);<br>Median (IQR)	85.8 (50.1)<br>73.0 (64.0 – 91.0)	71.3 (43.1)<br>60.0 (40.0 – 88.0)	102.4 (87.8)<br>62.0 (35.0 – 140.0)	0.58
Time to CPR board placement/rigid surface positioning from recognition of CA (s), M (SD);<br>Median (IQR)	41.0 (44.6)<br>33.0 (22.0 – 55.0)	28.4 (19.5)<br>18.0 (16.0 – 45.0)	73.9 (81.2)<br>37.0 (16.0 – 110.0)	0.18
Time to start ventilation (s), M (SD);<br>Median (IQR)	69.5 (27.6)<br>61.5 (55.0 – 74.5)	75.7 (30.3)<br>68.0 (55.0 – 90.0)	68.9 (27.9)<br>62.0 (47.0 – 83.0)	0.56
Time to start ventilation from recognition of CA (s), M (SD);<br>Median (IQR)	24.9 (26.0)<br>24.5 (12.5 – 36.0)	22.9 (32.0)<br>26.0 (10.0 – 37.0)	25.2 (28.0)<br>21.0 (15.0 – 33.0)	0.94
Time to IV/IO access placement (s), M (SD);<br>Median (IQR)	125.7 (42.9)<br>127.5 (96.0 – 157.0)	130.7 (66.3)<br>104.0 (85.0 – 177.0)	127.7 (60.6)<br>112.0 (84.0 – 176.0)	0.94
Time to IV/IO access placement from recognition of CA (s), M (SD);<br>Median (IQR)	81.4 (44.2)<br>80.5 (56.0 – 112.5)	78.2 (70.3)<br>70.0 (26.0 – 110.0)	83.8 (69.8)<br>77.0 (40.0 – 138.0)	0.94
Time to administration of first epinephrine (s), M (SD);<br>Median (IQR)	222.4 (63.8)<br>210.0 (192.5 – 233.0)	224.7 (48.0)<br>216.0 (191.0 – 261.0)	223.5 (67.1)<br>197.0 (182.0 – 253.0)	0.99
Time to administration of first epinephrine from recognition of CA (s), M (SD);<br>Median (IQR)	176.9 (61.4)<br>170.0 (149.5 – 188.5)	174.8 (51.6)<br>177.0 (147.0 – 206.0)	182.8 (69.2)<br>163.0 (136.0 – 222.0)	0.86
Time to administration of second epinephrine (s), M (SD);<br>Median (IQR)	417.4 (59.8)<br>400.0 (378.0 – 462.0)	422.3 (83.0)<br>415.0 (362.0 – 481.0)	401.6 (93.4)<br>406.0 (340.0 – 453.0)	0.56
Time to administration of second epinephrine from recognition of CA (s), M (SD);<br>Median (IQR)	372.1 (56.8)<br>368.0 (337.0 – 408.0)	369.7 (83.8)<br>358.0 (298.0 – 430.0)	364.2 (96.3)<br>362.0 (310.0 – 420.0)	0.93
Time to call for blood gas (s), M (SD);<br>Median (IQR)	289.9 (113.6)<br>273.5 (219.0 – 316.0)	330.4 (154.0)<br>330.0 (223.0 – 456.0)	342.3 (162.5)<br>303.0 (225.0 – 480.0)	0.35
Time to call for blood glucose (s), M (SD);<br>Median (IQR)	350.8 (120.3)<br>370.0 (267.0 – 429.0)	414.8 (118.0)<br>417.5 (366.0 – 487.0)	387.7 (148.0)<br>390.5 (293.0 – 524.0)	0.29

© 2023 Corazza F et al. *JAMA Network Open.*

Time to consider advanced airway management (s), M (SD);<br>Median (IQR)	265.3 (102.5)<br>238.0 (185.0 – 337.0)	306.4 (139.0)<br>274.5 (204.0 – 429.5)	289.8 (151.3)<br>265.0 (146.0 – 370.0)	0.62
Time to ask for history (s), M (SD);<br>Median (IQR)	297.5 (99.7)<br>254.0 (227.5 – 381.5)	414.9 (159.8)<br>454.5 (333.0 – 538.5)	385.5 (117.2)<br>387.5 (326.0 – 459.5)	**0.01**[d]
Time to treatment of hypovolemia (s), M (SD);<br>Median (IQR)	365.3 (114.5)<br>365.5 (288.0 – 448.0)	290.3 (134.5)<br>283.5 (216.0 – 361.0)	303.1 (131.7)<br>277.5 (218.0 – 322.0)	0.09
Time to treatment of hypoglycemia (s), M (SD);<br>Median (IQR)	408.6 (84.6)<br>417.5 (340.0 – 470.0)	458.7 (111.4)<br>481.0 (393.0 – 534.0)	422.9 (120.8)<br>438.0 (292.5 – 500.5)	0.43
Time to achievement of ROSC (s), M (SD);<br>Median (IQR)	465.6 (68.1)<br>457.0 (410.0 – 540.0)	469.2 (60.3)<br>453.5 (420.0 – 517.0)	470.7 (73.1)<br>472.5 (394.0 – 520.0)	0.98

Abbreviations: CA= cardiac arrest, n= number of teams, CPR= cardiopulmonary resuscitation, EKG= electrocardiogram, M= mean; IO= intraosseous, IQR= interquartile range, IV= intravenous, PALS= Pediatric Advanced Life Support, ROSC= Return of Spontaneous Circulation, s= seconds, SD= Standard Deviation, %= percentage.
Note: Pairwise comparisons (p value):
[a]PediaAppRREST vs PALS p=0.07, PediAppRREST vs null control p=0.97, PALS vs null control p=0.04;
[b]PediAppRREST vs PALS **p<0.001**, PediAppRREST vs null control **p<0.001**, PALS vs null control p=0.93;
[c]PediaAppRREST vs PALS **p<0.001**, PediAppRREST vs null control **p<0.001**, PALS vs null control p=0.90;
[d]PediAppRREST vs PALS **p=0.01**, PediAppRREST vs null control p=0.06, PALS vs null control p=0.76.

© 2023 Corazza F et al. *JAMA Network Open.*

eTable 5. Single Items of Clinical Performance Tool

CPT items	PediAppRREST (n=32)	PALS (n=35)	Null control (n=33)	P value
Item 1. Pulse check				0.77
Item 1. Score 0, n (%)	3 (9.4)	3 (8.6)	3 (9.1)	
Item 1. Score 1, n (%)	11 (34.4)	16 (45.7)	13 (39.4)	
Item 1. Score 2, n (%)	18 (56.3)	16 (45.7)	17 (51.5)	
Item 2. CPR				0.97
Item 2. Score 0, n (%)	0 (0.0)	0 (0.0)	0 (0.0)	
Item 2. Score 1, n (%)	7 (21.9)	8 (22.9)	19 (24.2)	
Item 2. Score 2, n (%)	25 (78.1)	27 (77.1)	14 (75.8)	
Item 3. EKG				0.49
Item 3. Score 0, n (%)	0 (0.0)	0 (0.0)	0 (0.0)	
Item 3. Score 1, n (%)	20 (62.5)	25 (71.4)	19 (57.6)	
Item 3. Score 2, n (%)	12 (37.5)	10 (28.6)	14 (42.4)	
Item 4. IV/IO access				0.20
Item 4. Score 0, n (%)	2 (6.3)	9 (25.7)	5 (15.2)	
Item 4. Score 1, n (%)	28 (87.5)	24 (68.6)	26 (78.8)	
Item 4. Score 2, n (%)	2 (6.3)	2 (5.7)	2 (6.1)	
Item 5. Epinephrine				0.38
Item 5. Score 0, n (%)	0 (0.0)	4 (11.4)	3 (9.1)	
Item 5. Score 1, n (%)	30 (93.8)	28 (80.0)	29 (87.9)	
Item 5. Score 2, n (%)	2 (6.3)	3 (8.6)	1 (3.0)	
Item 6. Pulse recheck after ROSC				**<0.001**[a]
Item 6. Score 0, n (%)	9 (28.1)	28 (80.0)	27 (81.8)	
Item 6. Score 1, n (%)	7 (21.9)	2 (5.7)	3 (9.1)	
Item 6. Score 2, n (%)	16 (50.0)	5 (14.3)	3 (9.1)	
Item 7. Defibrillation				0.46
Item 7. Score 0, n (%)	1 (3.1)	1 (2.9)	0 (0.0)	
Item 7. Score 1, n (%)	31 (96.9)	34 (97.1)	33 (100.0)	

Abbreviations: CPT= Clinical Performance Tool, CPR= cardiopulmonary resuscitation, EKG= electrocardiogram, IO= intraosseous, IV= intravenous, M= mean, n= number of teams, PALS= Pediatric Advanced Life Support, ROSC= Return of Spontaneous Circulation, SD= Standard Deviation, %= percentage.

Note: Pairwise comparisons: [a]PediAppRREST vs PALS **p<0.001**, PediAppRREST vs null control **p<0.001**, PALS vs null control p=0.96.

© 2023 Corazza F et al. *JAMA Network Open.*

eTable 6. Issues in Using the PediAppRREST App and Proposed Solutions

**Participants' issue in using the app**	**Number of teams**	**Proposed solution**
Distraction from the scenario	15	To choose a team member for the use of the app, different from the team leader
Conflict with the own mental order of priorities/actions or necessity of watching the guideline flow-chart/algorithm	11	Add the flow charts in the app
Reduction of the interaction with the rest of the team	7	To choose a team member for the use of the app, different from the team leader. A bigger screen to show the app to the rest of the team
Difficulties in using the reversible causes section	4	To make prompts for reversible causes always present
Difficulties in the familiarization with the app	3	Increase the time for familiarization and training with the app and the tablet
Low fidelity in the app and in the electronic cognitive aid	2	Increase the time for familiarization and training with the app and the tablet
Crash of the app	2	Solve the bugs and create some backups (i.e. stopwatch, an alternative action log, calculator)
Difficulty in managing advanced airway	2	Add a table of sizes for LMA/ETT for age/weight
Problems to unblock and use the tablet	1	To increase the familiarization and training with the app. To choose a team member for the use of the app, different from the team leader
Difficulties in preparing drugs	1	Anticipate the dosage of drugs before the administration
Difficulties in managing ROSC	1	Prompt the dose of drugs also in the ROSC section
Bug timer	1	Solve the bug
Too many countdowns	1	Combine rhythm and drug countdowns
Lost the actions made	1	Add a real time action log

Abbreviations: ETT= endotracheal tube; LMA= laryngeal mask airway; ROSC= Return of spontaneous circulation.

© 2023 Corazza F et al. *JAMA Network Open.*

eTable 7. Single Items of Raw NASA Task Load Index

R-TLX items	PediAppRREST (n=32)	PALS (n=34)*	Null control (n=33)	P value
Mental demand item, M (SD)	70.0 (20.9)	81.3 (14.7)	80.5 (13.4)	**0.011[a]**
Physical demand item, M (SD); Median (IQR)	16.3 (23.4)<br>7.5 (0.0 – 20-0)	15.3 (18.6)<br>10.0 (0.0 – 30.0)	16.7 (18.0)<br>10.0 (0.0 – 25.0)	0.96
Temporal demand item, M (SD); Median (IQR)	73.3 (19.7)<br>75.0 (67.5 – 85.0)	71.8 (18.9)<br>80.0 (55.0 – 85.0)	69.7 (22.2)<br>75.0 (60.0 – 85.0)	0.78
Performance item, M (SD)	58.1 (27.9)	54.0 (25.3)	60.0 (30.0)	0.66
Effort item, M (SD)	73.0 (18.6)	76.0 (17.6)	73.0 (17.1)	0.72
Frustration item, M (SD); Median (IQR)	58.0 (26.7)<br>55.0 (40.0 – 80.0)	62.6 (30.2)<br>77.5 (35.0 – 85.0)	67.7 (27.2)<br>70.0 (50.0 – 90.0)	0.38

Abbreviations: IQR= interquartile range, M= mean, min= minutes, n= number of teams, PALS= Pediatric Advanced Life Support, R-TLX= Raw NASA Task Load Index, SD= Standard Deviation, %= percentage.

Note: *data missing. Pairwise comparisons: [a]PediAppRREST vs PALS **p=0.02**, PediAppRREST vs null control **p=0.03**, PALS vs null control p=0.98.

© 2023 Corazza F et al. *JAMA Network Open.*
