# Supplementary material for: Effectiveness of a Novel Tablet Application in Reducing Guideline Deviations During Pediatric Cardiac Arrest: A Randomized Clinical Trial
Source: JAMA Netw Open. 2023 Aug 3;6(8):e2327272. doi: 10.1001/jamanetworkopen.2023.27272 (PMC10401301; doi:10.1001/jamanetworkopen.2023.27272)
Supplement: Supplement 3. — Data Sharing Statement [file jamanetwopen-e2327272-s003.pdf]

Data Sharing Statement

Corazza. Effectiveness of a Novel Tablet Application in Reducing Guideline Deviations During Pediatric Cardiac Arrest. *JAMA Netw Open*. Published August 03, 2023.
doi:10.1001/jamanetworkopen.2023.27272

Data
Data available: Yes
Data types: Deidentified participant data
How to access data: contact with silvia.bressan.1@unipd.it
When available: With publication

Supporting Documents
Document types: Statistical/analytic code
How to access documents: contact with annachiara.frigo@unipd.it
When available: With publication

Additional Information
Who can access the data: researchers whose proposed use of the data has been approved
Types of analyses: for specified purposes
Mechanisms of data availability: with investigator support after a signed data agreement
